# Microglia‐derived Galectin‐9 drives amyloid‐β pathology in Alzheimer's disease

**DOI:** 10.1111/acel.14396

**Published:** 2024-11-01

**Authors:** Guoxin Zhang, Qinyu Peng, Xiaodi Guo, Lina Pan, Min Xiong, Xingyu Zhang, Lijun Dai, Zhaohui Zhang, Tingting Xiao, Juanfeng He, Miao Liu, Wei Ke, Zhentao Zhang

**Affiliations:** ^1^ Department of Neurology Renmin Hospital of Wuhan University Wuhan China; ^2^ TaiKang Center for Life and Medical Sciences Wuhan University Wuhan China

**Keywords:** Alzheimer's disease, amyloid‐β, cross‐seeding, galectin‐9, neuroinflammation

## Abstract

The accumulation of amyloid‐β (Aβ) and overactivation of microglia contribute to the pathogenesis of Alzheimer's disease (AD), but the interaction between microglial activation and Aβ deposition in AD remains elusive. Here we revealed that Aβ activates microglia and promotes the release of Galectin‐9 (Gal‐9), a member of the β‐galactoside–binding family of lectins. The levels of Gal‐9 in the cerebrospinal fluid and brain tissues of AD patients are higher than those in control subjects. Gal‐9 interacts with Aβ and promotes its aggregation, generating Gal‐9‐Aβ fibrils with enhanced seeding activity and neurotoxicity. The expression of Gal‐9 increases with age in the brains of APP/PS1 transgenic mice. Knockout of Gal‐9 in APP/PS1 mice substantially reduced Aβ sedimentation, neuroinflammation, and cognitive impairment. Moreover, depletion of Gal‐9 inhibited the seeding activity of brain homogenates from APP/PS1 mice. These findings reveal a mechanism by which microglia‐derived Gal‐9 accelerates Aβ aggregation and seeding in AD. Thus, strategies aimed at inhibiting Gal‐9 may hold promise as a disease‐modifying therapy to alleviate AD pathology.

## Graphical abstract

We reported that Galectin‐9, highly expressed in the brains of AD patients and APP/PS1 mice, interacts with Aβ and generates Gal‐9‐Aβ fibrils with enhanced seeding activity and neurotoxicity. Gal‐9‐Aβ fibrils enhance Aβ‐induced microglial activation and promote Aβ pathology. Knockout of Galectin‐9 substantially attenuates Aβ pathology and cognitive impairment in APP/PS1 mice. Thus, strategies aimed at inhibiting Galectin‐9 could hold promise as a disease‐modifying therapy to alleviate Aβ pathology.

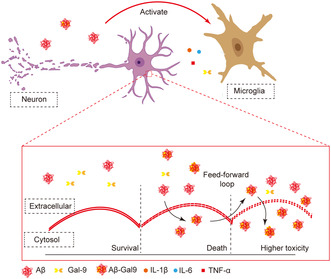

**Abbreviations**
Aβ: Amyloid‐β
AD: Alzheimer's disease
BiFC: Bimolecular fluorescence complementation
CNS: central nervous system
CRDs: carbohydrate recognition domains
CSF: cerebrospinal fluid
CTF: C‐terminal fragments
fEPSPs: field excitatory postsynaptic potentials
Gal‐9 KO: Gal‐9 knockout
Gal‐9: galectin‐9
IDE: insulin‐degrading enzyme
LTP: long‐term potentiation
NEP: Aβ‐degrading enzyme neprilysin
PFA: paraformaldehyde
TEM: transmission electron microscopy
ThioS: thioflavin S
ThT: thioflavin T
TX‐100: Triton X‐100
WT: wild‐type

## 1 INTRODUCTION

Alzheimer's disease (AD) is the most common cause of dementia. It is an age‐related progressive neurodegenerative disorder. Neuropathological hallmarks of AD include the deposition of extracellular amyloid‐β (Aβ) plaques, the formation of intracellular neurofibrillary tangles composed of hyperphosphorylated tau, and neuroinflammation (Kinney et al., 2018). The aggregation of Aβ has long been recognized to trigger the neurodegenerative cascade in AD (Karran & De Strooper, 2022). Aβ aggregates act as seeds for aggregate growth, inducing the propagation and spread of pathology in the brain. Microglia are the main resident immune cells and the main source of pro‐inflammatory molecules in the central nervous system (CNS) (Chen & Holtzman, 2022). Chronically activated microglia release a variety of pro‐inflammatory cytokines, inducing neuroinflammation and neurodegeneration (Baik et al., 2016). Microglia have been reported to regulate Aβ deposition (Guldner & Wyss‐Coray, 2023; Sarlus & Heneka, 2017), but the precise mechanisms are not fully understood.

Galectins are a family of glycan‐binding proteins that are involved in various biological functions (Marino et al., 2023). Recently, galectins have gained increasing recognition for their ability to regulate immune responses in neurological diseases (Vasta, 2009). Galectin family members modulate microglial polarization, immunosurveillance, and neuroinflammation (da Rosa et al., 2021). They play either complementary or antagonistic roles in neurodegenerative diseases (Yang et al., 2008). Gal‐1 has been found to inhibit microglial activation, restrict brain inflammation, and attenuate inflammation‐induced neurodegeneration (Nonaka & Fukuda, 2012; Parikh et al., 2015; Starossom et al., 2012). Conversely, Gal‐3 acts as an endogenous TREM2 ligand and detrimentally regulates the inflammatory response and promotes cognitive dysfunction (Boza‐Serrano et al., 2019, 2022; Tao et al., 2020). Gal‐9 is the most highly expressed galectin in the brain and is expressed and secreted mainly by microglia (John & Mishra, 2016). It regulates natural and adaptive immune responses, neuroinflammation, and the integrity of the blood–brain barrier (Duan et al., 2021; Steelman & Li, 2014; van Kooyk & Rabinovich, 2008). Interestingly, the levels of Gal‐9 in the cerebrospinal fluid (CSF) are positively associated with cognitive decline in HIV‐infected individuals (Premeaux et al., 2019). Furthermore, the levels of Gal‐9 in the CSF of AD patients are greater than those in control subjects and are positively correlated with cognitive impairment (Wang et al., 2019). Here we investigated the role of Gal‐9 in Aβ deposition and cognitive impairment. We found that Gal‐9 is highly expressed in the brains of AD patients and colocalizes with Aβ plaques. Gal‐9 interacts with Aβ and promotes its aggregation. Gal‐9 also enhances the seeding activity and neurotoxicity of Aβ fibrils. Gal‐9 deficiency ameliorates Aβ deposition and cognitive dysfunction in APP/PS1 mice. Thus, our study indicates that Gal‐9 plays a role in promoting Aβ deposition and cognitive impairment.

## 2 MATERIALS AND METHODS

### 2.1 Mice

Human *APP*_swe_ and *PSEN1*_dE9_ transgenic mice (APP/PS1 mice) were obtained from the Jackson Laboratory (stock number 005864). Gal‐9 knockout (Gal‐9 KO) mice were prepared by Cyagen Biosciences (Suzhou, China). They were both on the C57BL/6 background. Gal‐9 KO mice were constructed using CRISPR/Cas9 technology. The gRNA was designed and constructed for the gene fragment between exon 3 and 5 of the target gene LGALS9. The gRNA and Cas9 plasmids were co‐injected into fertilized eggs to obtain mice that do not express Gal‐9 in all cells in the body. Since Aβ pathology has been shown to vary between male and female mice (Kommaddi et al., 2023; Xiong et al., 2022), to minimize potential sex discrepancies in AD‐like pathology, only male mice were used in this study. The mice were housed under standard conditions at 22°C with a 12–12 h light–dark cycle with free access to food and water. Animal care and handling were performed according to the Declaration of Helsinki. All the animals were randomly assigned to different groups. The sample size was determined by Power and Precision (Biostat). Investigators were blinded to the group allocation during the animal experiments. The protocol was reviewed and approved by the Animal Care and Use Committee of Renmin Hospital, Wuhan University (WDRM20201118A).

### 2.2 Genotyping

For genotyping, the mouse tails were cut and lysed for DNA extraction and served as the template for PCR by the One Step Mouse Genotyping Kit (Vazyme). The genotyping primers for the transgenic APP were 5′‐AGGACTGACCACTCGACCAG‐3′ (transgene forward), 5′‐CGGGGGTCTAGTTCTGCAT‐3′ (transgene reverse), 5′‐CTAGGCCACAGAATTGAAAGATCT‐3′ (internal positive control forward) and 5′‐GTAGGTGGAAATTCTAGCATCATCC‐3′ (internal positive control reverse). The genotyping primers for transgenic PS1 were 5′‐AATAGAGAACGGCAGGAGCA‐3′ (transgene forward), 5′‐GCCATGAGGGCACTAATCAT‐3′ (transgene reverse), 5′‐CTAGGCCACAGAATTGAAAGATCT‐3′ (internal positive control forward) and 5′‐GTAGGTGGAAATTCTAGCATCATCC‐3′ (internal positive control reverse). The genotyping primers for Gal‐9 were 5′‐TACTTATGCCCACGTATAGCTGTC‐3′ (common forward), 5′‐GTGCAACTCCATTGTCATAGTTGG‐3′ (wild‐type reverse), and 5′‐GTGCTCTGTTGTGCCTTATGGG‐3′ (mutant reverse). The PCR products were separated by electrophoresis on a 1.5% agarose gel for genotype identification.

### 2.3 Human tissue samples

All brain tissues were obtained from the Emory Alzheimer's Disease Research Center (ADRC) Brain Bank. Human postmortem tissues were acquired under proper Institutional Review Board (IRB) protocols. AD was diagnosed according to the criteria of the Consortium to Establish a Registry for AD and the National Institute on Aging. Diagnoses were further confirmed by the presence of amyloid plaques and neurofibrillary tangles in formalin‐fixed tissue. The ages and postmortem times were similar between AD patients and controls. Informed consent was obtained from the subjects. The study was approved by the Biospecimen Committee.

### 2.4 ELISA quantification of CSF Gal‐9

CSF samples were collected from AD patients and age‐matched healthy controls via lumbar puncture. The study was approved by the ethics committee of Renmin Hospital, Wuhan University. Informed consent was obtained from all the subjects. The CSF was centrifuged, aliquoted, and stored at −80°C. Quantitative determination of Gal‐9 in CSF was performed via a sandwich ELISA Kit (Mlbio, ML038444V). Briefly, 50 μL of CSF sample was added to each well of a 96‐well microtiter plate pre‐coated with anti‐Gal‐9 antibody and incubated for 1 h at room temperature. After washing the plates were washed 5 times with PBST (0.05% Tween‐20 in PBS), 100 μL of HRP‐conjugated detection antibody was added, and the mixture was incubated for 1 h at room temperature. After washing, 100 μL of substrate was added to each well. The signals were measured on a microplate reader (Molecular Devices).

### 2.5 Quantification of cerebral Aβ_42_

The human Aβ_42_ solid‐phase sandwich ELISA Kit (Thermo Fisher, KHB3441) was used to determine the concentrations of Aβ_42_ in the brain tissues of the transgenic mice. Briefly, brain tissues from the hippocampus and cortex were homogenized with 8 volumes of cold 5 M guanidine‐HCl in 50 mM Tris, pH 8.0. The lysates were diluted 10 times with cold PBS supplemented with protease inhibitor cocktail and then centrifuged at 20,000 × *g* for 20 min at 4°C. Subsequently, 50 μL samples were added to the wells and incubated with detection antibody solution for 3 h at room temperature. The wells were subsequently washed with washing buffer and incubated with anti‐rabbit IgG HRP for 1 h at room temperature. The wells were washed with washing buffer. The stabilized chromogen was added and incubated for 30 min at room temperature in the dark. The signals were measured on a microplate reader (Molecular Devices).

### 2.6 Fibrillization of Aβ_42_ and ThT assay

Synthetic Aβ_42_ peptide was prepared by Qiangyao Biological Technology (Shanghai, China). To induce Aβ_42_ fibrillization, the Aβ_42_ peptide was dissolved in hexafluoroisopropanol to reach a final concentration of 1 mg/mL and sonicated on ice for 2 min to obtain a clear and colorless solution. The solution was placed in a fume hood overnight to obtain a thin and near‐transparent Aβ film. The film was dissolved in 50 μL of DMSO to form a colorless transparent solution. Then, PBS was added to obtain a 250 μM Aβ_42_ solution. To monitor Aβ_42_ fibrillization, a thioflavin T (ThT) fluorescence assay was performed as described previously (Kumar et al., 2011). Briefly, 10 μL of 2 mM ThT working solution was mixed with 90 μL of 50 μM Aβ_42_ solution in each microplate well. The fluorescence intensity was measured at excitation and emission wavelengths of 440 nm and 485 nm, respectively, with a slit width of 5 nm on a microplate reader (Molecular Devices). Recombinant human Gal‐9 protein (with an N‐terminal Fc tag) with a purity >90% was purchased from Atagenix Laboratories (catalog number: ATMP00560HU), as determined by SDS–PAGE quantitative densitometry after Coomassie blue staining. Recombinant Gal‐9 was dissolved in PBS to a final concentration of 0.1 mg/mL. To assess the effects of cross‐seeding on Aβ_42_ fibrillization, freshly prepared Aβ_42_ peptides (50 μM) were incubated with Gal‐9 peptides (0.4 μM and 0.8 μM) with or without an anti‐Gal‐9 neutralizing antibody at 37°C.

### 2.7 GST pull‐down assay

Plasmids encoding the GST‐Vector, GST‐Gal‐9, GST‐tagged N‐terminal or C‐terminal CRD of Gal‐9 were transfected into HEK293 cells stably transfected with GFP‐Aβ_42_. Forty‐eight hours later, the cells were collected and lysed in cell lysis buffer. The lysates were subsequently incubated with glutathione agarose beads (Thermo Fisher) overnight at 4°C for 4 h. The beads were subsequently washed 4 times in PBS with 0.1% Triton X‐100 and boiled in SDS loading buffer. The binding between Gal‐9 and Aβ_42_ was then measured by Western blotting.

### 2.8 Bimolecular fluorescence complementation (BiFC)

BiFC assays were performed as described previously (Kerppola, 2006). The Venus fluorescence protein was selected as the reporter for complementation. Human Aβ_42_‐ and Gal‐9‐coding sequences were cloned and inserted into EcoRI‐ and KpnI‐digested pBiFC‐VC155 and pBiFC‐VN173 plasmids, respectively. HEK293T cells co‐transfected with pBiFC‐VC155‐Aβ_42_ and pBiFC‐VN173‐Gal‐9 for 48 h were analyzed. Vector plasmids of pBiFC‐VC155 and pBiFC‐VN173 were used as negative controls.

### 2.9 Real‐time RT‐PCR

Total mRNA was isolated from BV2 cells via TRIzol reagent (Invitrogen). Complementary DNA was synthesized via an iScript cDNA synthesis kit (Bio‐Rad). Quantitative PCR was performed on a LightCycler 480 real‐time PCR system using LightCycler 480 SYBR Green 1 Master Mix (Roche). The following primers were used: mouse Gal‐9 forward primer (5′‐ATGCCCTTTGAGCTTTGCTTC‐3′) and reverse primer (5′‐AACTGGACTGGCTGAGAGAAC‐3′); GAPDH forward primer (5′‐AGGTCGGTGTGAACGGATTTG‐3′) and reverse primer (5′‐TGTAGACCATGTAGTTGAGGTCA‐3′). The relative gene expression was normalized to GAPDH expression and assessed via the 2^−ΔΔCt^ method.

### 2.10 Western blot

Mouse brain tissue or cultured cells were lysed for 30 min at 4°C in lysis buffer (50 mM Tris [pH 7.4], 150 mM NaCl, 1 mM EDTA, 1% Triton X‐100, 0.1% sodium dodecyl sulfate, 50 mM NaF, 10 mM sodium pyrophosphate, and 10 mM sodium β‐glycerophosphate supplemented with protease inhibitor cocktail) and centrifuged for 30 min at 15000 rpm at 4°C. The protein concentrations in the supernatants were determined with a BCA protein assay kit (Thermo Fisher). After protein separation by 8%–12% SDS–PAGE, the samples were transferred to a nitrocellulose membrane. The membranes were blocked with 5% nonfat milk in TBS containing 0.1% Tween 20 (TBST) and then incubated with primary antibodies overnight at 4°C. The membranes were washed 3 times in TBST and incubated with horseradish peroxidase (HRP)‐conjugated anti‐mouse or anti‐rabbit antibodies for 1 h at room temperature. Immunoreactivity was visualized via enhanced chemiluminescence (ECL) via an enhanced chemiluminescence (ECL) Western blotting system (Bio‐Rad). Primary antibodies against the following targets were used: β‐tubulin (Proteintech, 10,068‐1‐AP, 1:10,000), β‐actin (Proteintech, 20,536‐1‐AP, 1:10,000), GST (Proteintech, 66,001‐2‐Ig, 1:1000), GFP (Proteintech, HRP‐66002, 1:1000), APP and C‐terminal cleavage fragments α‐CTF, β‐CTF (Abcam, ab32136, 1:2000), IBA1 (Proteintech, 10,904‐1‐AP, 1:1000), GFAP (Proteintech, 60,190‐1‐Ig, 1:2000), PSD95 (Cell Signaling Technology, 3409 s, 1:2000), Synapsin I (Cell Signaling Technology, 2312 s, 1:2000), Synaptophysin (Cell Signaling Technology, 36,406 s, 1:2000), ADAM10 (Proteintech, 25,900‐1‐AP, 1:2000), BACE‐1 (Cell Signaling Technology, 5606, 1:2000), IDE (Proteintech, 67,106‐1‐Ig, 1:2000), and NEP (Proteintech, 18,008‐1‐AP, 1:2000).

### 2.11 Immunohistochemistry and immunofluorescence

The mice were anesthetized and transcardially perfused with cold PBS and then 4% paraformaldehyde (PFA). The brains were subsequently fixed for 24 h in 4% PFA at 4°C and then embedded in paraffin. Serial 5‐μm‐thick sections from all the animals were processed in parallel for immunohistochemistry and immunofluorescence. After blocking endogenous peroxidase activity with 3% H_2_O_2_ for 10 min and washing three times in PBS, the sections were incubated in PBS with 1% BSA and 0.3% Triton X‐100 for 30 min followed by overnight incubation with primary antibodies at 4°C. The sections were washed three times in PBS with 0.1% Triton X‐100. For the thioflavin S (ThioS) assay, the sections were stained with 1% ThioS for 5 min. For immunohistochemistry, the signal was developed with a Histostain‐SP Kit (Invitrogen). For immunofluorescence staining, the slices were incubated with secondary antibodies conjugated to Alexa Fluor 488 or Alexa Fluor 594. Primary antibodies against the following targets were used: Gal‐9 (Abcam, ab69630, 1:1000), CD31 (ImmunoWay, YM4916, 1:1000), Aβ (BioLegend, 6E10, sig‐39,300, 1:1000), MAP2 (Proteintech, 17,490‐1‐AP, 1:2000), and IBA1 (Wako, 019–19,741, 1:1000).

### 2.12 Primary neuron culture

Primary cortical neurons were derived from wild‐type (WT) mice at embryonic day 18 and cultured in Neurobasal medium supplemented with B27 supplement. Aβ, Gal‐9, and Gal‐9‐Aβ were added to the culture medium (5 μg/mL) at 7 days in vitro (DIV). Six days later, the neurons were fixed, permeabilized, and immunostained with TUNEL BrightRed Apoptosis Detection Kit (Vazyme) and subjected to Hoechst/PI staining. The sections were examined under a fluorescence microscope (IX73, Olympus).

### 2.13 Primary microglia culture

Primary mixed glial cultures were prepared from the cerebral cortex of neonatal WT or Gal‐9 ko mice (P0‐P2). After 10–15 days in culture, microglia were isolated by mechanical shaking (400 rpm, 1 h) and purified by plating on sterile dishes. Microglia were cultured in Dulbecco's modified Eagle's medium (DMEM) supplemented with 10% fetal bovine serum.

### 2.14 CCK‐8 assay

SH‐SY5Y cells were seeded in 96‐well plates at a density of 1 × 10^4^/100 μL. PBS, Gal‐9, Aβ, or Gal‐9‐Aβ was added to the medium, and the mixture was incubated for 24 h at 37°C in a 5% CO_2_ incubator. Ten microliters of CCK‐8 solution was added to each well. The cells were incubated for 4 h at 37°C. The absorbance at 460 nm was determined via a microplate reader (Molecular Devices).

### 2.15 Tissue extracts

Mouse brain homogenates (cortex and hippocampus) were prepared from WT, Gal‐9 KO, APP/PS1, and APP/PS1;Gal‐9 KO mice (12 months old) following the previously described method (Venegas et al., 2017). Brain tissue samples were lysed for 30 min at 4°C in lysis buffer (50 mM Tris [pH 7.4], 150 mM NaCl, 1 mM EDTA, 1% Triton X‐100, 0.1% sodium dodecyl sulfate, 50 mM NaF, 10 mM sodium pyrophosphate, and 10 mM sodium β‐glycerophosphate supplemented with protease inhibitor cocktail) and centrifuged for 30 min at 15000 rpm at 4°C. ELISA was used to adjust for equal amounts of Aβ in aliquots of brain homogenates from APP/PS1 and APP/PS1;Gal‐9 KO mice by the addition of WT mouse brain homogenate. Aliquots were stored at −80°C before use.

Three‐month‐old mice subjected to stereotaxic surgery were anesthetized with isoflurane and placed into an automated stereotaxic instrument. The mice received a unilateral stereotaxic injection of (1) PBS, Gal‐9, Aβ fibrils, or Gal‐9‐Aβ fibrils; (2) brain extracts prepared from APP/PS1, APP/PS1;Gal‐9 KO, or WT mice; or (3) brain extracts prepared from APP/PS1 mice that were pre‐treated with anti‐Gal‐9‐IgG or isotype‐IgG via Hamilton syringes into the hippocampus at AP‐2.5 mm, L‐2.0 mm, and DV‐1.8 mm. Each mouse received 5 μL of protein mixture (5 μg) at a speed of 0.1 μL/min. The needle was kept in place for an additional 10 min before it was slowly withdrawn to avoid refluxing the needle tract. The mice were monitored until they fully recovered.

### 2.16 Morris water maze test

The Morris water maze test was used to detect the spatial learning and memory of the mice (Zhang et al., 2022; Zhang, Song, et al., 2014). The mice were trained for four trials per day for 5 consecutive days. The mice were allowed to search for the platform for 60 s. Otherwise, they were guided to the platform manually and allowed to stay on it for 15 s before the next trial. After all the trials, the mice were dried and returned to their cages. After the last training day (Day 5), a spatial probe trial was performed. The platform was removed, and the mice were allowed to swim for 60 s. The percentage of time spent in the platform quadrant was measured. All trials were recorded by a computerized tracking system that analyzed the distance the mice moved, the latency required to reach the platform, and the swimming speed via ANY‐Maze software (San Diego Instruments).

### 2.17 Y‐maze test

Three arms were randomly designated at a 120° angle from each other, including a start arm, a novel arm, and a third arm. During the first trial (training), the mice were allowed to explore the start arm and the third arm for 5 min while the novel arm was blocked. After a 1 h intertrial interval, the second trial (retention) was conducted, and the mice had free access to all three arms for 5 min. The number of entries and time spent in the new arm were recorded and analyzed with ANY‐Maze software (San Diego Instruments).

### 2.18 Electron microscopy of synapses

The synaptic structure and density were determined via electron microscopy. The mice were anesthetized and perfused transcardially with 2% glutaraldehyde. Then, the hippocampal slices were postfixed in 1% cold OsO_4_ for 1 h. Samples were prepared and examined via standard procedures. Ultrathin sections (90 nm) were stained with uranyl acetate and lead acetate and viewed at 100 kV under a JEOL 200CX transmission electron microscope (TEM). Synapses were identified by the presence of synaptic vesicles and postsynaptic densities. The number of synaptic clefts, synapse density, synaptic active zone length and width of the synaptic clefts in the hippocampal CA1 area were quantified via ImageJ software (NIH).

### 2.19 Golgi staining

Golgi staining was performed with an FD Rapid Golgi stain kit (FD Neuro Technologies, PK401) according to the manufacturer's instructions. Mouse brains were fixed in 10% formalin for 24 h and then immersed in 3% potassium bichromate for 3 days in the dark. The solution was changed every day. The brains were subsequently transferred to 2% silver nitrate solution and incubated for 24 h in the dark. Vibratome sections were cut at 60 μm, air‐dried for 10 min, dehydrated through 95% and 100% ethanol, cleared in xylene, and assembled on coverslips. Bright‐field images of pyramidal neurons in the hippocampus and cortex were taken at 100× magnification via a Zeiss Axioplan microscope (Zeiss, Decatur, GA, USA). To measure spine density, all clearly evaluable areas of 50–100 μm of secondary dendrites from the imaged neurons were counted and quantified via ImageJ software (NIH).

### 2.20 Electrophysiology

Acute hippocampal transverse slices were prepared from 12‐month‐old WT, Gal‐9 KO, APP/PS1 and APP/PS1; Gal‐9 KO mice. Briefly, the mice were deeply anesthetized, and the brains were rapidly removed and placed in ice‐cold oxygenated cutting solution containing the following (in mM): 25 D‐glucose, 2.5 KCl, 1.25 NaH_2_PO_4_, 26 NaHCO_3_, 7.2 MgCl_2_, 0.5 CaCl_2_, 3.1 Na–pyruvate, 11.35 ascorbic acid and 97 choline chloride. (pH 7.4) at ~310 mOsm under 95% O2 and 5% CO_2_. Coronal slices (350 μm thick) containing the dorsal hippocampus were cut at 4°C in the cutting solution via a Leica VT1200S vibratome and then transferred to an incubation chamber filled with oxygenated artificial cerebrospinal fluid (aCSF), which contained the following: 118 mM NaCl, 2.5 mM KCl, 1 mM NaH2PO4, 26 mM NaHCO_3_, 2 mM MgCl_2_, 2 mM CaCl_2_, and 22 mM glucose, in a 35°C water bath for 30 min and then placed at room temperature for 30 min before being recorded. The brain slices were transferred to a chamber perfused with different recording bath solutions. For LTP recordings, a 0.1‐MΩ tungsten monopolar electrode was used to stimulate the Schaffer collaterals in the stratum radiatum. Field excitatory postsynaptic potentials (fEPSPs) were recorded in the CA1 stratum radiatum via a glass microelectrode filled with artificial CSF with a resistance of 3–4 MΩ. Field potential input–output curves were constructed by measuring fEPSP slopes as the stimulus intensity increased from 1 to 10 V, with a 0.5‐V increment. LTP of fEPSPs was induced by three theta bursts (TBS, 4 pulses at 100 Hz, repeated 3 times with a 200‐ms interval). The magnitudes of LTP are expressed as the mean percentage of the initial slope of the baseline fEPSP.

### 2.21 Statistical analysis

Statistical analysis was performed with GraphPad Prism 7.0 (GraphPad Prism, San Diego, CA, USA). Independent data were introduced into the statistical analysis (Aarts et al., 2014). The data are shown as means ± SEM. Statistical comparisons between two groups were performed with Student's *t*‐tests. For analysis of more than two groups, one‐way or two‐way ANOVA was performed followed by post hoc analysis where appropriate. Differences with *p* < 0.05 were considered significant.

## 3 RESULTS

### 3.1 Gal‐9 is highly expressed in the brains of AD patients and APP/PS1 mice

**FIGURE 1:**
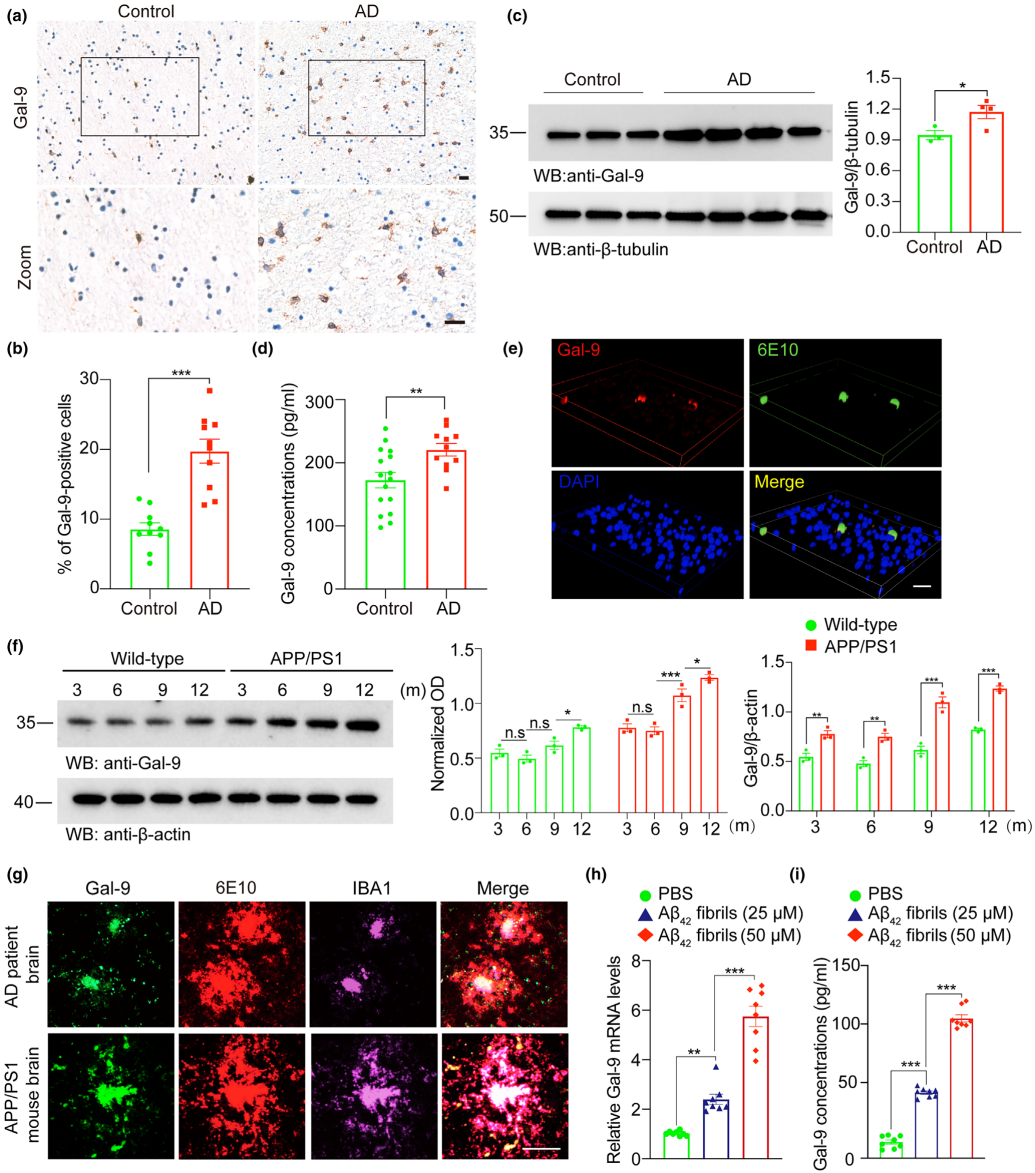
Gal‐9 expression in the brains of AD patients and APP/PS1 mice. (a) Representative images of Gal‐9 expression in the hippocampus of AD patients and age‐matched control subjects. (b) Quantification of the percentage of Gal‐9‐positive cells. Scale bars, 20 μm. Bars represent means ± SEM. Unpaired Student's *t*‐test. *n* = 10 AD patients and 10 control subjects (one slide from each subject). ***p < 0.001. (c) Western blot analysis of Gal‐9 in the hippocampus of AD patients and age‐matched controls. *n* = 4 AD patients and 3 controls. Bars represent means ± SEM. Unpaired Student's *t*‐test. *p < 0.05. (d) Gal‐9 concentrations in the CSF of AD patients (*n* = 11) and healthy controls (*n* = 16). Bars represent means ± SEM. Unpaired Student's *t*‐test, **p < 0.01. (e) Co‐immunostaining of Gal‐9 and Aβ in brain sections from APP/PS1 mice. Scale bars, 20 μm. (f) Western blot analysis of Gal‐9 levels in the brains of APP/PS1 and WT mice at different ages. Bars represent means ± SEM. Unpaired Student's *t*‐test. **p < 0.01, ***p < 0.001. (g) Co‐immunostainings of Gal‐9, Aβ plaques, and microglia in the brains of AD patients (upper panel) and APP/PS1 mice (lower panel). Scale bars, 50 μm. (h) RT‐PCR analysis of Gal‐9 mRNA levels in BV2 cells exposed to Aβ fibrils. (i) ELISA analysis of Gal‐9 levels in the supernatant of BV2 cells exposed to Aβ fibrils. Bars represent means ± SEM. One‐way ANOVA followed by Tukey's post hoc test. **p < 0.01, ***p < 0.001.

To examine whether Gal‐9 participates in Aβ pathology in AD, we first immunostained Gal‐9 in hippocampal brain slices from AD patients and age‐matched healthy controls. The levels of Gal‐9 were much higher in the brain sections of AD patients than in those of control subjects (Figure 1a,b; Table S1). Western blot analysis confirmed the increased level of Gal‐9 in AD brains (Figure 1c). Furthermore, ELISA analysis revealed that Gal‐9 concentrations in the CSF of AD patients were greater than those in the CSF of control subjects (Figure 1d; Table S2). We further tested the expression of Gal‐9 in the brains of APP/PS1 transgenic mice and found that Gal‐9 was elevated in APP/PS1 mouse brains and colocalized with Aβ plaques (Figure 1e). Aging is the most important risk factor for AD. Thus, we further tested the levels of Gal‐9 in the brains of WT and APP/PS1 mice at different ages. The levels of Gal‐9 increased in an age‐dependent manner in both WT and APP/PS1 mice. APP/PS1 mice presented more severe increases in Gal‐9 (Figure 1f). These findings indicate that Gal‐9 expression in the brain is age‐dependent, with particularly elevated levels in the APP/PS1 mouse model.

Microglia are the main source of Gal‐9 in the brain (Zhang, Chen, et al., 2014). To determine the distribution of Gal‐9 in the AD brain, we co‐stained Gal‐9 with the microglial marker IBA1, and found that Gal‐9 predominantly colocalized with microglia and was especially confined to Aβ plaque‐associated microglia in AD patients and APP/PS1 mice (Figure 1g). To determine whether Aβ_42_ fibrils regulate Gal‐9 expression in vitro, we incubated BV2 cells with Aβ_42_ fibrils. RT‐PCR analysis revealed that exposure to Aβ_42_ fibrils increased Gal‐9 mRNA expression in BV2 cells (Figure 1h). ELISA confirmed that the levels of Gal‐9 in the culture medium of BV2 cells increased after exposure to Aβ_42_ fibrils (Figure 1i). Similar results were obtained in primary microglia (Figure S1a). Thus, Aβ fibrils increase the expression and secretion of Gal‐9 by BV2 microglia.

### 3.2 Gal‐9 interacts with Aβ and accelerates its aggregation in vitro

To determine whether Gal‐9 affects Aβ aggregation, recombinant Aβ_42_ monomers (50 μM) were incubated with or without Gal‐9 (0.81 μM). ThT fluorescence assay revealed that Aβ aggregation was markedly accelerated in a Gal‐9 concentration‐dependent manner in vitro (Figure S1b). In contrast, the scrambled polypeptide failed to affect Aβ_42_ aggregation (Figure S1b). Furthermore, the effect of Gal‐9 on Aβ_42_ aggregation was abolished in the presence of an anti‐Gal‐9 antibody (Figure S1c). We observed the morphology of the Aβ_42_ fibrils formed in the presence of Gal‐9 via transmission electron microscopy (TEM) and revealed that Gal‐9 altered the morphology of the Aβ_42_ fibrils (Figure S1d). These results indicate that Gal‐9 accelerates Aβ_42_ fibrillization and results in the formation of Gal‐9‐Aβ fibrils with morphologies distinct from those of Aβ_42_ fibrils.

To investigate whether Gal‐9 specifically interacts with Aβ_42_ in vitro, we transfected HEK293 cells with plasmids carrying the GST‐vector or GST‐Gal‐9 and then co‐incubated the cell extracts with Aβ_42_ oligomers. GST pull‐down analysis revealed that Gal‐9 interacts with Aβ_42_ oligomers (Figure S1e). Furthermore, we co‐transfected HEK293 cells with plasmids encoding GST‐Gal‐9 and GFP‐Aβ_42_. GST pull‐down assay confirmed the interaction between Gal‐9 and Aβ_42_ (Figure S1f). To further investigate whether Gal‐9 affects Aβ_42_ aggregation in cells, we transfected GST‐Gal‐9 or GST‐vector plasmids into HEK293 cells stably transfected with GFP‐Aβ_42_. Strikingly, after permeabilization with Triton X‐100 (TX‐100) to eliminate soluble Aβ_42_, abundant insoluble Aβ_42_ aggregates were present in cells overexpressing Gal‐9 but not in control cells (Figure S2a), indicating that Gal‐9 is sufficient to trigger Aβ_42_ fibrilization in vitro. Finally, we confirmed the interaction between Gal‐9 and Aβ_42_ via the pBiFC assay, a technique used to validate protein interactions in living cells (Kerppola, 2006). Strong fluorescent signals were observed in HEK293 cells that were co‐transfected with plasmids encoding pBiFC‐VN173‐Aβ_42_ and pBiFC‐VC155‐Gal‐9 (Figure S2b), confirming that Gal‐9 and Aβ_42_ interact with each other.

Gal‐9 contains two carbohydrate recognition domains (CRDs) in the N‐ and C‐terminal regions connected by a linking peptide. To investigate which CRD of Gal‐9 interacts with Aβ_42_, we performed GST pull‐down assay with GST‐tagged Gal‐9 fragments and revealed that the C‐terminal CRD but not the N‐terminal CRD of Gal‐9 interacts with Aβ_42_ (Figure S2c). Consistently, when the N‐ and C‐terminal CRDs were overexpressed in HEK293 cells stably transfected with GPF‐Aβ_42_, only the C‐terminal CRD, not the N‐terminal CRD, induced Aβ_42_ aggregation (Figure S2d). Thus, Gal‐9 interacts with Aβ_42_ and accelerates its aggregation through its C‐terminal CRD.

### 3.3 Gal‐9 enhances Aβ‐induced microglial activation and neurotoxicity in vitro

To investigate whether Gal‐9 affects the properties of Aβ fibrils, we challenged BV2 cells with equal amounts of Gal‐9, Aβ fibrils, or Gal‐9‐Aβ fibrils. RT‐PCR and ELISA analysis revealed that the mRNA levels of pro‐inflammatory cytokines, including interleukin‐1β (IL‐1β), interleukin‐6 (IL‐6), and tumor necrosis factor‐α (TNF‐α), were elevated in cells exposed to Gal‐9 or Aβ fibrils. Interestingly, the Gal‐9‐Aβ fibrils had the strongest effect on increasing the mRNA and protein levels of IL‐1β, IL‐6, and TNF‐α (Figure S3a,b). To assess the effect of the Gal‐9 antibody, we treated BV2 cells with the antibody and stimulated them with recombinant Gal‐9 protein. Gal‐9 antibody blocked the impact of Gal‐9 on microglial activation (Figure S3c). Thus, this antibody functions as a neutralizing antibody. Next, we tested the effects of Aβ and Gal‐9‐Aβ fibrils on primary cortical neurons. After the neurons were exposed to Gal‐9, Aβ fibrils, or Gal‐9‐Aβ fibrils, TUNEL assays revealed that, compared with pure Aβ fibrils, Gal‐9‐Aβ fibrils induced more neuronal apoptosis (Figure S3d,e). These results were confirmed by CCK‐8 assay in SH‐SY5Y cells (Figure S3f) and Hoechst/PI staining of neurons (Figure S4a,b). Furthermore, we cultured primary microglia from WT and Gal‐9 KO mouse brains and treated them with Aβ_42_ fibrils. The conditioned medium was collected and added to primary neurons and SH‐SY5Y cells. TUNEL and CCK‐8 assays revealed greater neurotoxic effects of CM from WT microglia than from Gal‐9 KO microglia (Figure S4c–f). Together, these results indicate that Gal‐9‐Aβ is more potent in inducing microglial inflammation and neuronal toxicity.

### 3.4 Gal‐9‐Aβ fibrils induce more severe motor impairments than Aβ fibrils do in APP/PS1 mice

**FIGURE 2:**
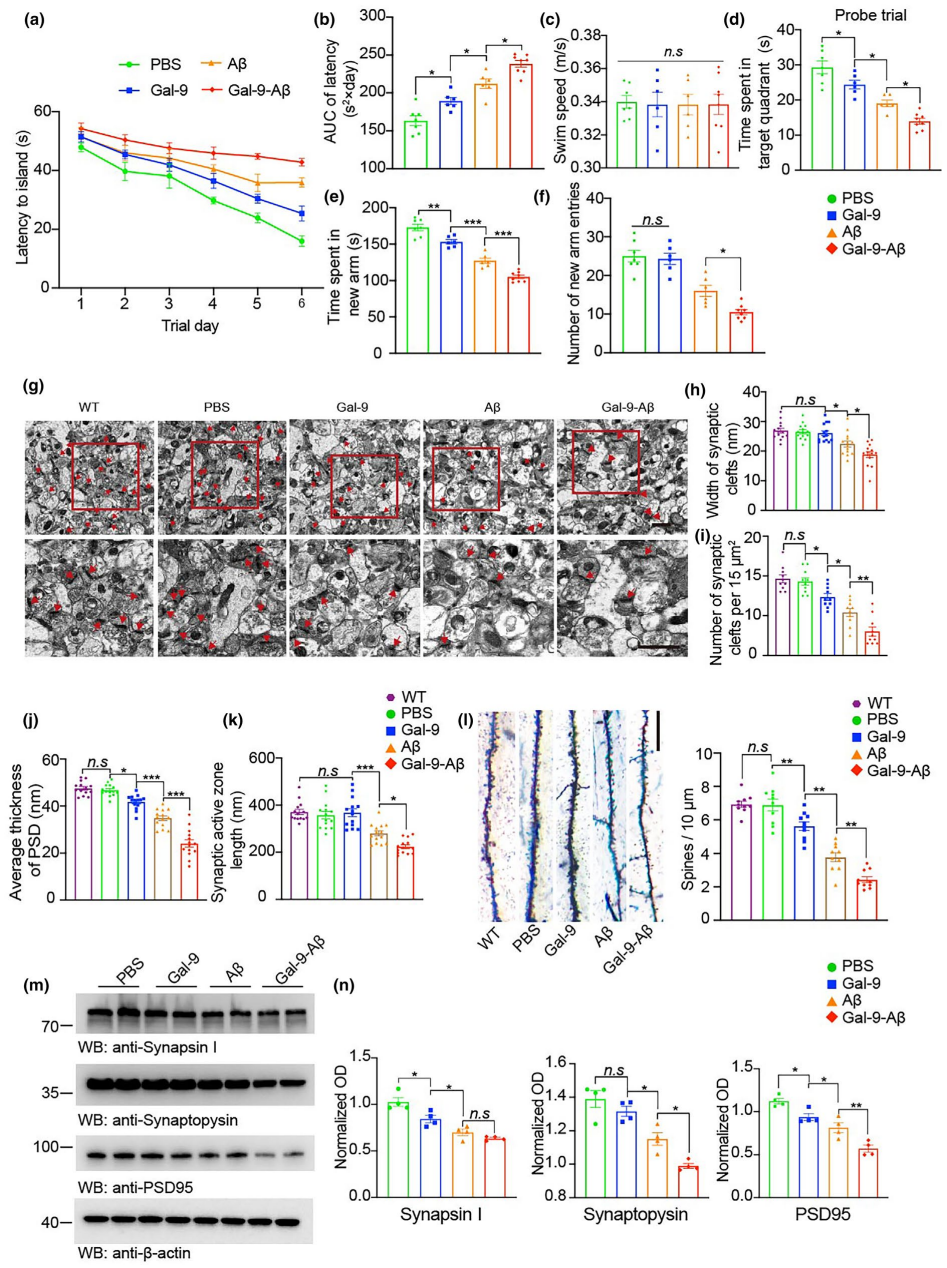
Gal‐9‐Aβ fibrils induce cognitive impairment and synaptic degeneration in APP/PS1 mice. Three‐month‐old male mice were injected with PBS, Gal‐9, Aβ, or Gal‐9‐Aβ. The mice were sacrificed 6 months after the operation. Spatial memory was assessed in the Morris water maze test (a–d) and Y maze test (e, f). (a) The latency to the platform in the Morris water maze test. (b) Integrated time traveled in the Morris water maze test. AUC, area under the curve. (c) The average swim speed of mice. (d) Time spent in the target quadrant in the probe trial. (e, f) Y‐maze test results. Shown are the time spent in the new arm (e) and the number of new arm entries (f). *n* = 7 mice in the PBS group, *n =* 6 mice in the Gal‐9 group, *n =* 6 mice in the Aβ group, *n* = 8 mice in the Gal‐9‐Aβ group. (g) Electron microscopy of synapses (top) and magnified images (below). Scale bar, 1 μm. (h) Width of synaptic clefts. (i) The number of synaptic clefts. (j) Postsynaptic density. (k) Length of the active zone. *n* = 14 slices from 4 different mice per group. (l) Golgi staining of dendritic spines of hippocampal slices. Scale bar, 15 μm. *n* = 10 slices from 4 different mice per group. (m, n) Representative immunoblots (m) and quantification (n) of synapsin I, synaptophysin, and PSD‐95 in the hippocampus of mice. *n* = 4 mice per group. Data are presented as means ± SEM. One‐way ANOVA followed by Tukey's post hoc test. n.s, not significant, *p < 0.05, **p < 0.01, ***p < 0.001.

To explore whether Gal‐9 enhances the neurotoxicity of Aβ in vivo, we injected Aβ fibrils or Gal‐9‐Aβ fibrils into the unilateral hippocampus of three‐month‐old APP/PS1 transgenic mice. The protein concentrations of the Aβ or Gal‐9‐Aβ fibrils were normalized before injection. Special learning and memory were assessed via the Morris water maze test and Y‐maze test at 6 months post‐injection. The results revealed that the mice injected with Aβ or Gal‐9‐Aβ fibrils exhibited cognitive decline, with Gal‐9‐Aβ fibrils showing a stronger detrimental effect than Aβ fibrils (Figure 2a–f). Consistent with the results of the behavioral tests, electron microscopy revealed more severe synaptic degeneration in the Gal‐9‐Aβ group than in the Aβ group (Figure 2g–k). Furthermore, Golgi staining revealed a substantial reduction in the number of dendritic spines in the Gal‐9‐Aβ group (Figure 2l). Interestingly, compared with age‐matched WT mice, APP/PS1 mice presented comparable synaptic and cognitive functions, as demonstrated by electron microscopy of synapses and Golgi staining (Figure 2g–l). These data suggest that synaptic and cognitive functions remain intact in 9‐month‐old APP/PS1 mice but are impaired by treatment with Gal‐9, Aβ, or a combination of Gal‐9. Moreover, immunoblot analysis of brain homogenates from hippocampal sections revealed a substantial reduction in the levels of synaptic proteins, including synapsin I, synaptophysin, and PSD95, in the Gal‐9‐Aβ group (Figure 2m,n). These results indicate that, compared with Aβ, Gal‐9‐Aβ induces more severe synaptic dysfunction and cognitive impairments in APP/PS1 mice.

### 3.5 Gal‐9‐Aβ fibrils induce increased Aβ pathology and neuroinflammation

**FIGURE 3:**
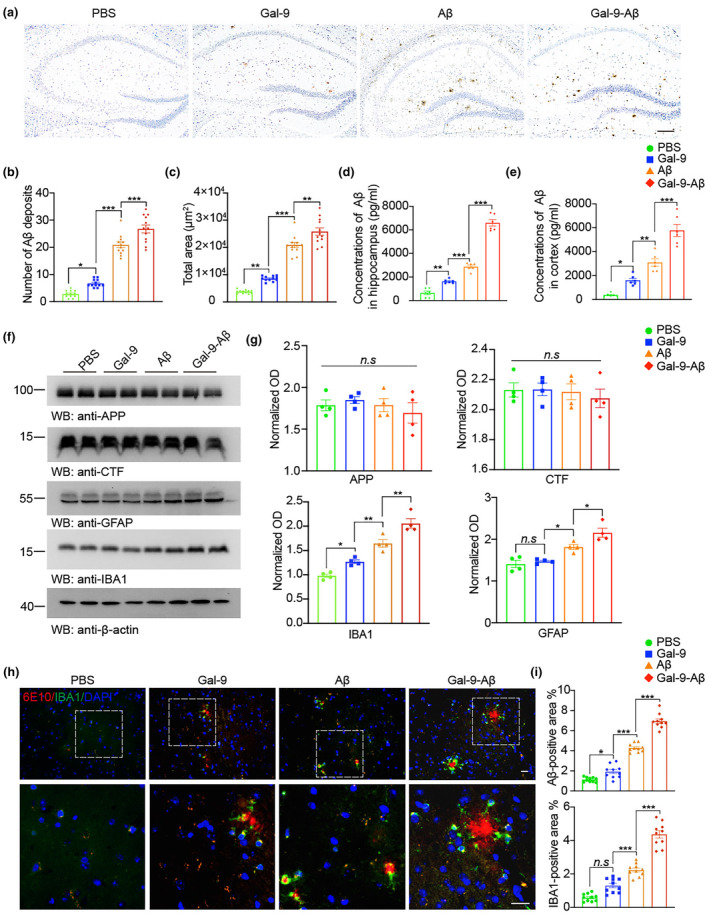
Gal‐9‐Aβ fibrils accelerate Aβ deposition and induce neuroinflammation in APP/PS1 mice. Three‐month‐old male mice were injected with PBS, Gal‐9, Aβ, or Gal‐9‐Aβ. The mice were sacrificed 6 months after the operation. (a) Representative images of Aβ deposits in the hippocampus of APP/PS1 mice. Scale bar, 200 μm. (b, c) Quantification of the number (b) and area (c) of Aβ deposits. *n* = 12 slices from 4 mice per group. ELISA of Aβ concentrations in the hippocampus (d) and cortex (e) in APP/PS1 mice. *n* = 6 samples from 3 mice per group. (f, g) Representative immunoblots and quantification of APP, α‐ and β‐C‐terminal fragments (α‐CTF and β‐CTF), IBA‐1, and GFAP in the hippocampal lysates. *n* = 4 mice per group. Data are presented as means ± SEM. One‐way ANOVA followed by Tukey's post hoc test. n.s, not significant, *p < 0.05, **p < 0.01, ***p < 0.001. (h, i) Co‐immunostaining and quantification of Aβ and the microglial markers IBA1 in the cortex of APP/PS1 mice. Scale bar, 20 μm. *n* = 10 slices from 4 mice per group. Data are presented as means ± SEM. One‐way ANOVA followed by Tukey's post hoc test. n.s, not significant, *p < 0.05, ***p < 0.001.

To investigate whether Gal‐9 enhances the seeding activity of Aβ in vivo, we detected Aβ deposits in APP/PS1 mice that received unilateral hippocampal injections of PBS, Gal‐9, Aβ fibrils and Gal‐9‐Aβ fibrils. Six months after injection, we found that, compared with the injection of pure Aβ fibrils, the injection of Gal‐9‐Aβ fibrils significantly increased the number and total area of Aβ deposits in the hippocampus (Figure 3a–c). These results were confirmed by ThioS staining (Figure S5a‐d). ELISA revealed that the levels of Aβ in the hippocampus and cortex were greater in the mice injected with the Gal‐9‐Aβ fibrils than in those injected with the Aβ fibrils (Figure 3d,e). No change was observed in the levels of APP and APP cleavage products in the different groups (Figure 3f,g). These results indicate that, compared with pure Aβ fibrils, Gal‐9‐Aβ fibrils are more potent at inducing Aβ aggregation. Microglia‐mediated neuroinflammation contributes to neurodegeneration in AD. Thus, we further investigated the effect of Gal‐9‐Aβ fibrils on the activation of microglia and astrocytes. Western blot analysis revealed that the levels of the microglial marker IBA1 and the astrocyte marker GFAP were much greater in the mice injected with the Gal‐9‐Aβ fibrils than in those injected with the Aβ fibrils (Figure 3f,g). Injection of Gal‐9‐Aβ fibrils also increased the density of IBA‐1‐positive cells in the brain slices (Figure 3h,i; Figure S5e,f).

### 3.6 Deletion of Gal‐9 attenuates Aβ deposition and neuroinflammation in APP/PS1 mice

**FIGURE 4:**
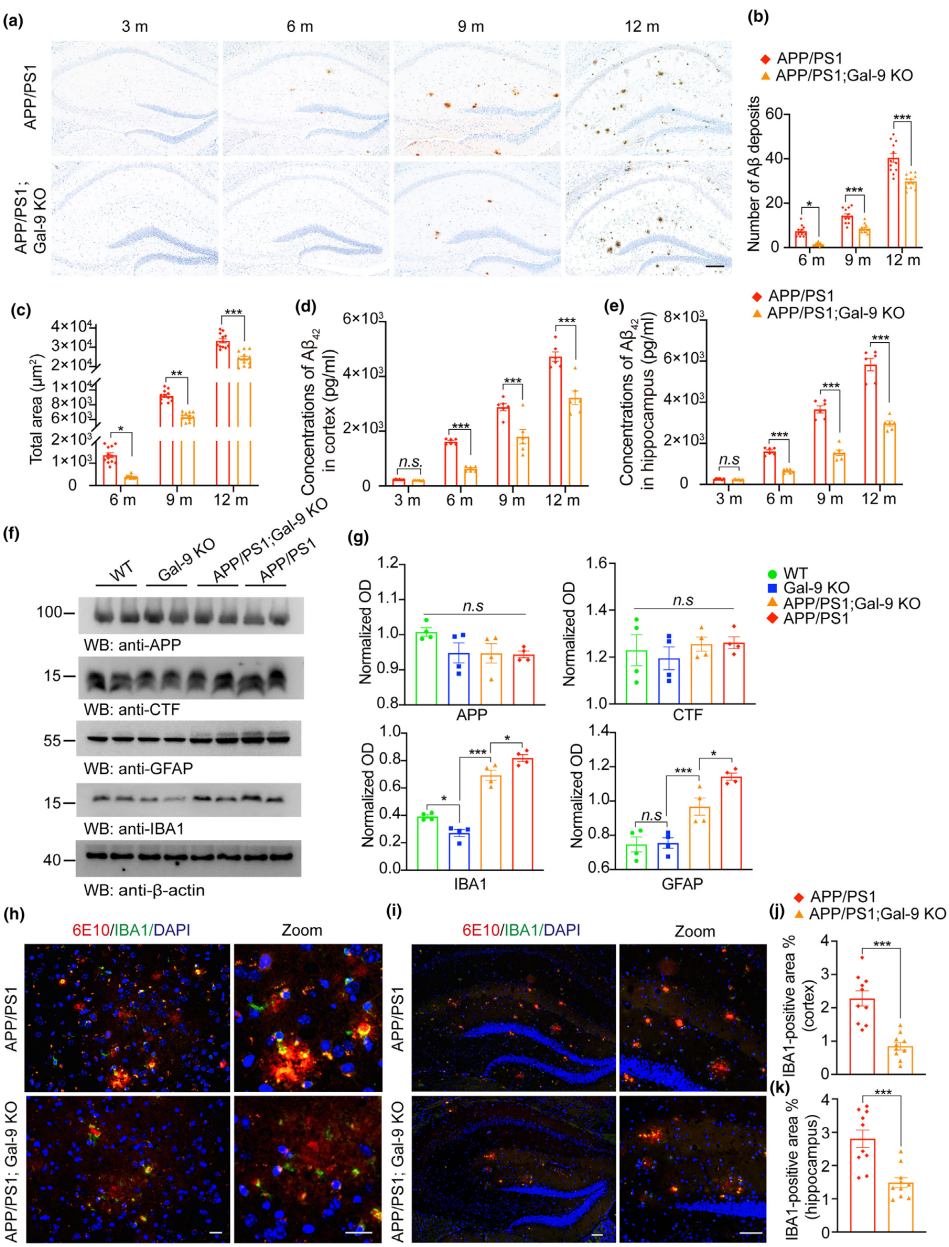
Gal‐9 deficiency rescues cognitive decline in APP/PS1 mice. WT, Gal‐9 KO, APP/PS1 and APP/PS1;Gal‐9 KO mice were sacrificed at 3, 6, 9, or 12 months old. (a) Representative images of Aβ deposits in the mouse hippocampus. (b, c) Quantification of the area (b) and number (c) of Aβ deposits in the hippocampus of APP/PS1 and APP/PS1;Gal‐9 KO mice. *n* = 12 mice. (d, e) ELISA analysis of Aβ_42_ concentrations in the cortex (d) and hippocampus (e) in APP/PS1 and APP/PS1;Gal‐9 KO mice. *n* = 6 mice per group. (f, g) Brain lysates were immunoblotted (f) and quantified (g) for APP, α‐ and β‐C‐terminal fragments (α‐CTF and β‐CTF), IBA‐1, and GFAP in the hippocampus of mice. *n* = 4 mice per group. Data are presented as means ± SEM. One‐way ANOVA followed by Tukey's post hoc test. n.s, not significant, *p < 0.05, **p < 0.01, ***p < 0.001. (h–k) Immunostaining and quantification of Aβ and the microglial markers IBA1 and in the cortex (h, j) and hippocampus (i, k) of 10‐month‐old APP/PS1 and APP/PS1;Gal‐9 KO mice in (a). Scale bar, 20 μm in the cortex; 100 μm in the hippocampus. *n* = 10 slices from 4 mice per group. Data are presented as means ± SEM. One‐way ANOVA followed by Tukey's post hoc test. ***p < 0.001.

To further characterize the role of Gal‐9 in Aβ pathology and cognitive impairment, APP/PS1 mice were crossed with Gal‐9 KO mice to generate APP/PS1;Gal‐9 KO mice. In addition to microglia, Gal‐9 is also present in endothelial cells and may regulate angiogenesis. To investigate the effect of Gal‐9 on angiogenesis, we first examined its expression in endothelial cells. Immunofluorescence revealed that Gal‐9 does not co‐localize with CD31‐positive endothelial cells (Figure S6a). To further assess the impact of Gal‐9 knockout on angiogenesis, we stained CD31 on brain tissue from WT, Gal‐9 KO, APP/PS1, and APP/PS1;Gal‐9 KO mice. The results demonstrated that the knockout of Gal‐9 did not alter angiogenesis in either WT or APP/PS1 mice (Figure S6b,c). To further explore the role of Gal‐9 in the context of Aβ pathology, four groups of mice were analyzed at 3, 6, 9 and 12 months of age: WT, Gal‐9 KO, APP/PS1, and APP/PS1;Gal‐9 KO mice. Aβ deposition was barely detectable in 3‐month‐old APP/PS1 and APP/PS1‐Gal‐9 KO mice. As the mice aged, Aβ deposition gradually increased in both groups. In 12‐month‐old mice, the density of Aβ aggregates was much lower in the hippocampi of APP/PS1;Gal‐9 KO mice than in those of APP/PS1 mice (Figure 4a–c). Similar results were obtained via ThioS staining (Figure S7a–f). Moreover, ELISA revealed substantially lower Aβ levels in the hippocampus and cortex of APP/PS1;Gal‐9 KO mice than in those of APP/PS1 mice (Figure 4d,e). The levels of APP and C‐terminal fragments of APP (α‐CTF, β‐CTF) were similar in APP/PS1 and APP/PS1;Gal‐9 KO mice (Figure 4f,g). Furthermore, there were no differences in the expression of ADAM10, BACE1, the Aβ‐degrading enzyme neprilysin (NEP), or insulin‐degrading enzyme (IDE) in the hippocampi of the mice (Figure S7g,h). Western blot analysis revealed that the levels of the microglial marker IBA1 and the astrocyte marker GFAP were greater in APP/PS1 mice than in WT and Gal‐9 KO mice, whereas APP/PS1;Gal‐9 KO mice presented decreased levels of IBA1 and GFAP compared with those in APP/PS1 mice (Figure 4f,g). Similarly, the density of IBA1‐positive cells was also lower in the cortex and hippocampus of APP/PS1;Gal‐9 KO mice than in those of APP/PS1 mice (Figure 4h–k). These results suggest that the deletion of Gal‐9 attenuates Aβ deposition and neuroinflammation.

### 3.7 Gal‐9 deficiency ameliorates memory deficits and synaptic dysfunction in APP/PS1 mice

**FIGURE 5:**
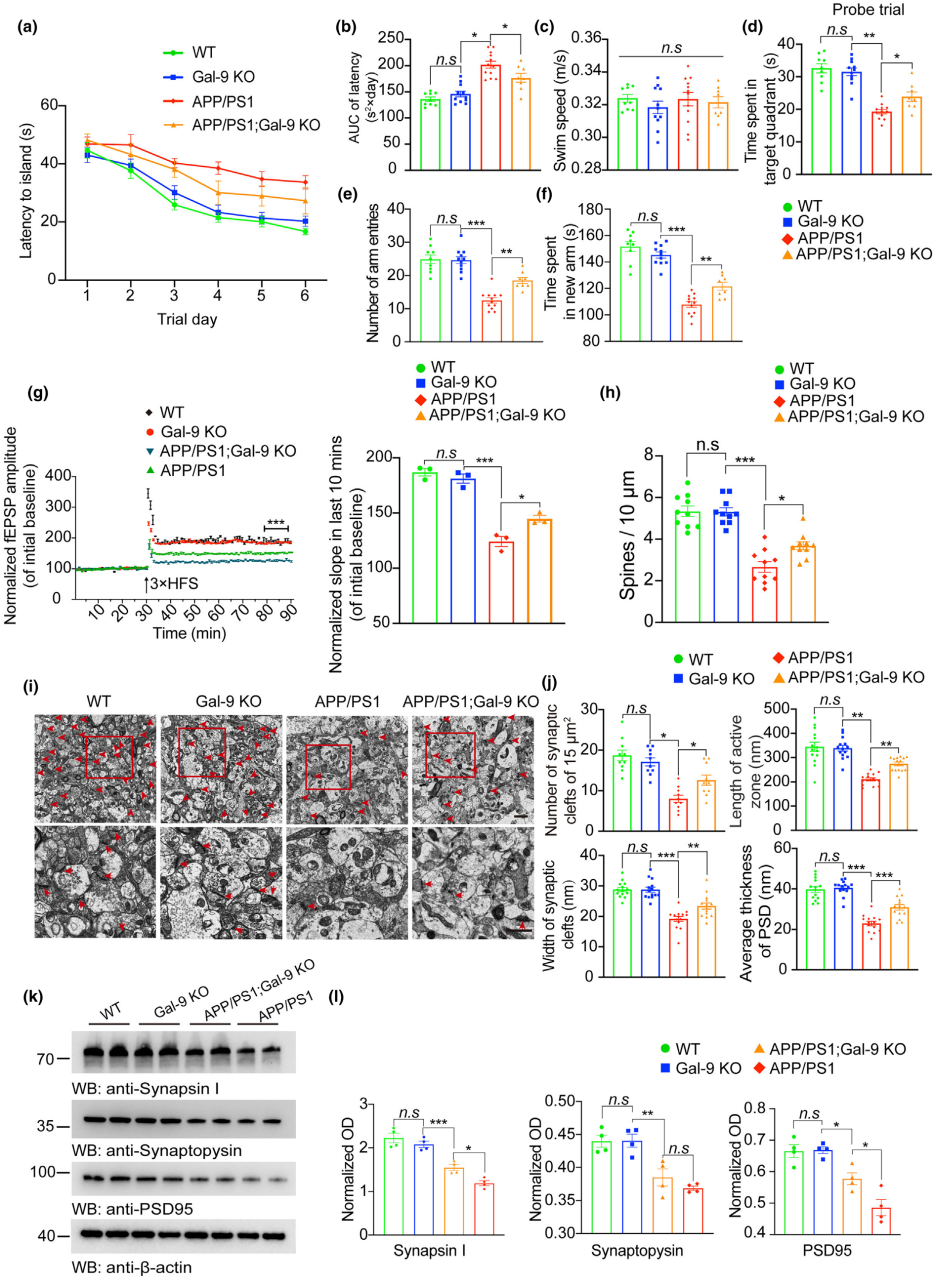
Gal‐9 deficiency rescues memory deficits and synaptic dysfunction in APP/PS1 mice. The spatial memory of WT, Gal‐9 KO, APP/PS1 and APP/PS1;Gal‐9 KO mice was assessed in the Morris water maze test and Y maze test. (a) The latency traveled to the platform. (b) Integrated time traveled in the Morris water maze test. AUC, area under the curve. (c) Average swim speed of mice. (d) Probe trial results. (e, f) Time spent in the new arm (e) and number of new arm entries (f) in the Y‐maze test. *n* = 9 mice in the WT group, *n =* 11 mice in the Gal‐9 KO group, *n =* 8 mice in the APP/PS1 group, *n* = 12 mice in the APP/PS1; Gal‐9 KO group. (g) Long‐term potentiation (LTP) of fEPSPs was induced by 3 × TBS (4 pulses at 100 Hz, repeated three times with a 200 ms interval). The magnitude of LTP and synaptic transmission was assessed by input/output (I/O). (h) Golgi staining and quantification of dendritic spines in hippocampal slices. Scale bar, 15 μm. *n* = 10 slices per group. (i) Electron microscopy of synapses (top) and magnification (bottom). Scale bar, 1 μm. (j) The number of synaptic clefts, length of active zone, width of synaptic clefts, and postsynaptic density of synapses. *n* = 14 slices from 4 mice per group. (k, l) Hippocampal lysates were immunoblotted for synapsin I, synaptophysin, and PSD95. *n* = 4. Data are presented as the means ± SEM. One‐way ANOVA followed by Tukey's post hoc test. n.s, not significant, *p < 0.05, **p < 0.01, ***p < 0.001.

Morris water maze test and Y maze test were performed to investigate the cognition of WT, Gal‐9 KO, APP/PS1, and APP/PS1; Gal‐9 KO mice. During the training phase, the swim distance and latency to find the platform progressively decreased in the WT and Gal‐9 KO mice, demonstrating a learning effect. Compared with WT and Gal‐9 KO mice, APP/PS1 mice presented learning deficits. Interestingly, compared with APP/PS1;Gal‐9 KO mice, APP/PS1;Gal‐9 KO mice presented substantially improved spatial memory performance (Figure 5a,b). The mice in the different groups displayed comparable swimming speeds, indicating no difference in motor function (Figure 5c). In the probe trial, APP/PS1;Gal‐9 KO mice performed better than APP/PS1 mice did in terms of memory retention, as illustrated by the greater percentage of time spent in the target quadrant (Figure 5d). Similarly, compared with APP/PS1 mice, APP/PS1;Gal‐9 KO mice presented attenuated learning deficits in spatial memory in the Y‐maze test (Figure 5e,f). These results indicate that Gal‐9 deficiency rescues AD‐like cognitive impairments in APP/PS1 mice.

We further evaluated the long‐term potentiation (LTP) of field excitatory postsynaptic potentials (fEPSPs) in the hippocampal CA1 region, which represents synaptic plasticity. LTP was diminished in APP/PS1 mice compared with WT mice (Figure 5g). LTP impairment was attenuated by the deletion of Gal‐9. Golgi staining revealed a substantial reduction in the density of dendritic spines in the APP/PS1 mice, but Gal‐9 deficiency reduced the loss of dendritic spines (Figure 5h). Consistently, electron microscopy revealed that the density of synapses was substantially reduced and that the synaptic structure was disrupted in the hippocampal CA1 region of APP/PS1 mice. Knockout of Gal‐9 attenuated the loss of dendritic spines and synapses (Figure 5i,j). Western blot analysis revealed that the deletion of Gal‐9 attenuated the reduction in the expression of synaptic proteins, including synapsin I, synaptophysin, and PSD95, in APP/PS1 mice (Figure 5k,l). These results indicate that Gal‐9 deficiency reduces synaptic dysfunction and rescues cognitive impairments in APP/PS1 mice.

### 3.8 Depletion of Gal‐9 reduces the seeding activity of Aβ aggregates

**FIGURE 6:**
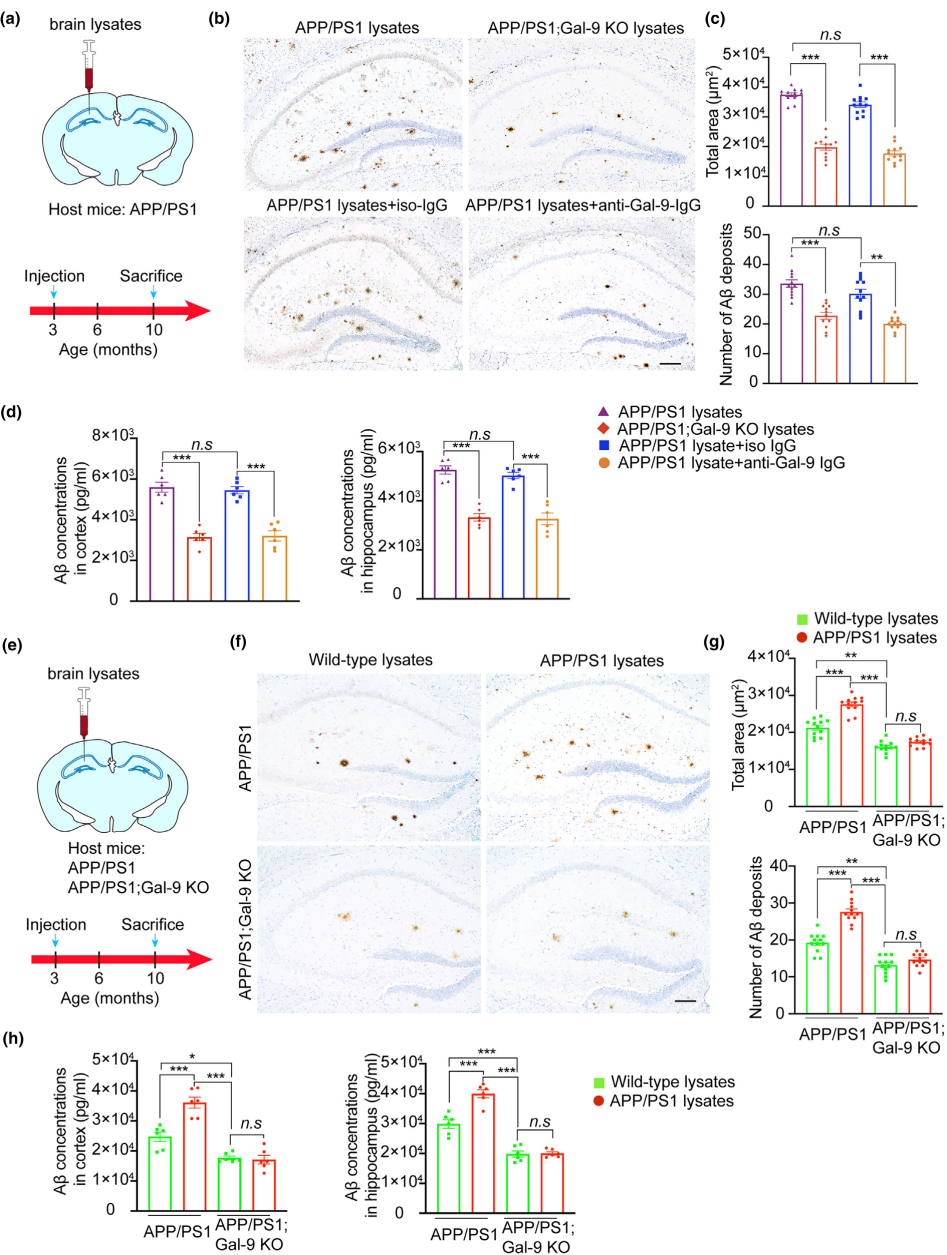
Blockade of Gal‐9 halts the progression of Aβ pathology. (a–d) Three‐month‐old APP/PS1 mice received unilateral intra‐hippocampal injections of: (1) brain homogenates derived from APP/PS1 mice, (2) brain homogenates derived from APP/PS1;Gal‐9 KO mice, (3) APP/PS1 mouse brain homogenates pre‐treated with anti‐Gal‐9 antibody, or (4) APP/PS1 mouse brain homogenates pre‐treated with iso‐IgG. (a) Schematic for brain lysate and antibody injection experiments. (b) Immunohistochemistry using 6E10 antibody showing Aβ deposits in the hippocampus. (c) Total Aβ‐positive area and number of Aβ plaques. *n* = 12 samples from 4 mice per group. (d) ELISA analysis of Aβ_42_ in the cortex and hippocampus. (e, f) APP/PS1 mice or APP/PS1; Gal‐9 KO mice received a unilateral injection of brain homogenates from APP/PS1 and WT mice, respectively. (e) Schematic for brain lysate injection experiments. (f) Representative immunohistochemistry images showing Aβ deposits in the hippocampus. (g) Quantification of the number and area of Aβ‐positive deposits in the hippocampus. (h) ELISA analysis of Aβ_42_ in the cortex and hippocampus. Scale bar, 200 μm. *n* = 12 slices from 4 mice per group. Data are presented as means ± SEM. Two‐way ANOVA followed by Tukey's post hoc test. n.s, not significant, **p < 0.01, ***p < 0.001.

To test whether Gal‐9 modulates the seeding activity of Aβ aggregates, we extracted insoluble brain homogenates from 12‐month‐old APP/PS1 mice and APP/PS1;Gal‐9 KO mice. The concentrations of Aβ in the brain homogenates were normalized to those in the WT brain lysates. The brain homogenates were injected into the hippocampi of 3‐month‐old APP/PS1 mice (Figure 6a). Seven months after injection, the number and area of Aβ plaques were greater in the APP/PS1 mice than in the APP/PS1;Gal‐9 KO mice (Figure 6b,c). ELISA revealed substantially lower Aβ concentrations in the hippocampus and cortex of APP/PS1 mice injected with APP/PS1;Gal‐9 KO brain homogenates than in those injected with APP/PS1 brain homogenates (Figure 6d).

To exclude the potential confounding factors responsible for Aβ spreading, we co‐incubated brain homogenates derived from APP/PS1 mice with a Gal‐9‐specific antibody or an isotype‐specific IgG and then injected them into the hippocampus of 3‐month‐old APP/PS1 mice (Figure 6a). Compared with those in the iso‐IgG group, the number and area of Aβ plaques in the Gal‐9 antibody group were reduced 7 months after injection (Figure 6b,c). Consistently, ELISA analysis revealed that the concentrations of Aβ in the brain of the Gal‐9 antibody group were lower than those in the iso‐IgG group (Figure 6d). Taken together, these results indicate that Gal‐9 is required for the efficient propagation of Aβ aggregates.

To further confirm the contribution of endogenous Gal‐9 to Aβ spreading, we prepared hippocampal homogenates from APP/PS1 mice and WT mice and injected them into the hippocampi of 3‐month‐old APP/PS1 or APP/PS1;Gal‐9 KO mice (Figure 6e). At 10 months of age, the APP/PS1 mice that received injections of APP/PS1 brain homogenates presented an increased number and area of Aβ‐positive deposits compared with those that received WT homogenates (Figure 6f,g). However, injection of APP/PS1 brain lysates failed to accelerate the progression of Aβ pathology in APP/PS1;Gal‐9 KO mice (Figure 6f,g). The concentrations of Aβ in the hippocampus and cortex of APP/PS1 mice that were injected with APP/PS1 brain homogenates were greater than those in APP/PS1;Gal‐9 KO mice that were injected with APP/PS1 brain homogenates (Figure 6h). Overall, these results demonstrate the important role of endogenous Gal‐9 in accelerating Aβ pathology in vivo.

## 4 DISCUSSION

The deposition of Aβ and overactivation of the innate immune system are hallmarks of AD pathology (Cisbani & Rivest, 2021). Neurotoxic Aβ fibrils activate microglia and promote the release of pro‐inflammatory mediators, which leads to neuronal injury. In this work, we found that Gal‐9 is highly expressed in the brains of AD patients and APP/PS1 model mice and that it colocalizes with Aβ plaques. Gal‐9 interacts with Aβ and accelerates its aggregation, generating Gal‐9‐Aβ fibrils, which induce neuroinflammation, synaptic dysfunction and cognitive decline, whereas the knockout of Gal‐9 rescues Aβ deposition, neuroinflammation and learning memory in APP/PS1 mice. These data suggest that Gal‐9 plays a pivotal role in Aβ deposition and AD progression.

Microglia are innate immune cells in the brain that regulate neuroinflammation (Voet et al., 2019). Given that neuroinflammation and Aβ deposition correlate with each other and contribute to neurodegeneration, deciphering the relationship between Aβ aggregation and neuroinflammation is critically important (Heneka et al., 2015; Kwon & Koh, 2020; Uddin et al., 2022). In the elderly brain, microglia are overactivated, which phagocytose Aβ and promote the production and release of pro‐inflammatory factors. Gal‐9 is the most highly expressed galectin in the brain and tends to aggregate (John & Mishra, 2016). There are reports that Gal‐9 can regulate microglial activation (Steelman & Li, 2014) and is highly related to cognitive dysfunction, not only in HIV‐related cognitive decline but also in AD patients (Wang et al., 2019). However, it remains unknown whether Gal‐9 is involved in Aβ pathology. Here, we show that the levels of Gal‐9 in the brain and CSF are greater in AD patients than in control subjects. Furthermore, Gal‐9 is spatially colocalized with Aβ plaques and plaque‐associated microglia in the brains of AD patients. This provides the spatial basis for Gal‐9 to interact with Aβ (Wang et al., 2019). Interestingly, Aβ treatment increased the expression and secretion of Gal‐9, indicating that Aβ aggregation and Gal‐9 secretion may form a vicious cycle that induces sustained neuroinflammation and neurodegeneration.

Many factors, such as ASC specks and amylin, have been shown to promote Aβ aggregation (Ly et al., 2021; Venegas et al., 2017). Here, we found that Gal‐9 promotes the fibrillization of Aβ. This effect was compromised by an anti‐Gal‐9 neutralizing antibody. Gal‐9 interacts with Aβ through its C‐terminal CDR. Aβ may aggregate into different strains under different conditions. Different Aβ strains show distinct seeding activities and neurotoxicity. This may explain the heterogeneity of AD patients (Qiang et al., 2017). Interestingly, Aβ fibrils formed in the presence of Gal‐9 presented increased aggregation ability and seeding activity and triggered more severe neuronal injury than pure Aβ fibrils in vitro. Furthermore, injection of Gal‐9‐Aβ fibrils into the hippocampus of APP/PS1 mice triggered more severe Aβ deposition, synaptic dysfunction, and cognitive impairment than injection of pure Aβ fibrils, indicating that Gal‐9‐seeded Aβ may represent a novel Aβ strain.

Galectin family members contain intrinsically disordered peptides that are prone to aggregation (Nieminen et al., 2007; Ono et al., 2014). To test whether endogenous Gal‐9 influences Aβ pathology, we crossed APP/PS1 and Gal‐9 KO mice to generate APP/PS1;Gal‐9 KO mice and found that Gal‐9 deficiency substantially reduced cognitive disability, reduced Aβ deposition and spreading, and attenuated synaptic damage. In addition, the mouse brain homogenates derived from APP/PS1;Gal‐9 KO mice presented decreased seeding activity compared with those from APP/PS1 mice, further suggesting that Gal‐9 is required for the efficient seeding of Aβ aggregates. These results suggest that exogenous Gal‐9 plays an important role in promoting Aβ‐related pathology.

Our findings indicate a bidirectional relationship between Gal‐9 and Aβ pathology. Specifically, Aβ stimulates the expression of Gal‐9. On the other hand, Gal‐9 promotes Aβ aggregation in a dose‐dependent manner, reinforcing the cycle of plaque formation and neurotoxicity in AD. This reciprocal interaction points to a potential causal role for Gal‐9 in exacerbating Aβ pathology. Gal‐9 may act as a key mediator in the amplification of Aβ‐related neurodegeneration. Furthermore, the ability of Gal‐9 to modulate microglial polarization may play a role, potentially driving pro‐inflammatory responses that contribute to synaptic dysfunction and cognitive decline.

In summary, the findings of our study position Gal‐9 as a significant player in the pathology of AD, particularly through its involvement in Aβ aggregation and its regulatory effects on neuroinflammation. Blocking Gal‐9 may be a novel therapeutic strategy to disrupt the cycle of Aβ accumulation and mitigate neurodegenerative processes. Understanding the causal relationships among Gal‐9, Aβ aggregation, microglia‐induced inflammation, and neurodegeneration may help uncover potential peripheral biomarkers for the early diagnosis of AD and the development of therapeutic strategies to alleviate neurodegeneration in AD.

## AUTHOR CONTRIBUTIONS

Z.Z. conceived the project and designed the experiments. G.Z. performed most of the experiments. X.G., Q.P., L.P., M.X., X.Z., and L.D. helped with the cell culture and animal experiments. Z.Z., T.X., J.H., M.L. and W.K. helped with the data analysis and interpretation.

## CONFLICT OF INTEREST STATEMENT

The authors report no competing interests.

## Supporting information


Figure S1.

Figure S2.

Figure S3.

Figure S4.

Figure S5.

Figure S6.

Figure S7.

Table S1.

Table S2.

## Data Availability

All data are available from the corresponding author upon reasonable request from any qualified investigator.
