# Supplementary figures and images for: Microglia‐derived Galectin‐9 drives amyloid‐β pathology in Alzheimer's disease
Source: Aging Cell. 2024 Nov 1;24(2):e14396. doi: 10.1111/acel.14396 (PMC11822670; doi:10.1111/acel.14396)

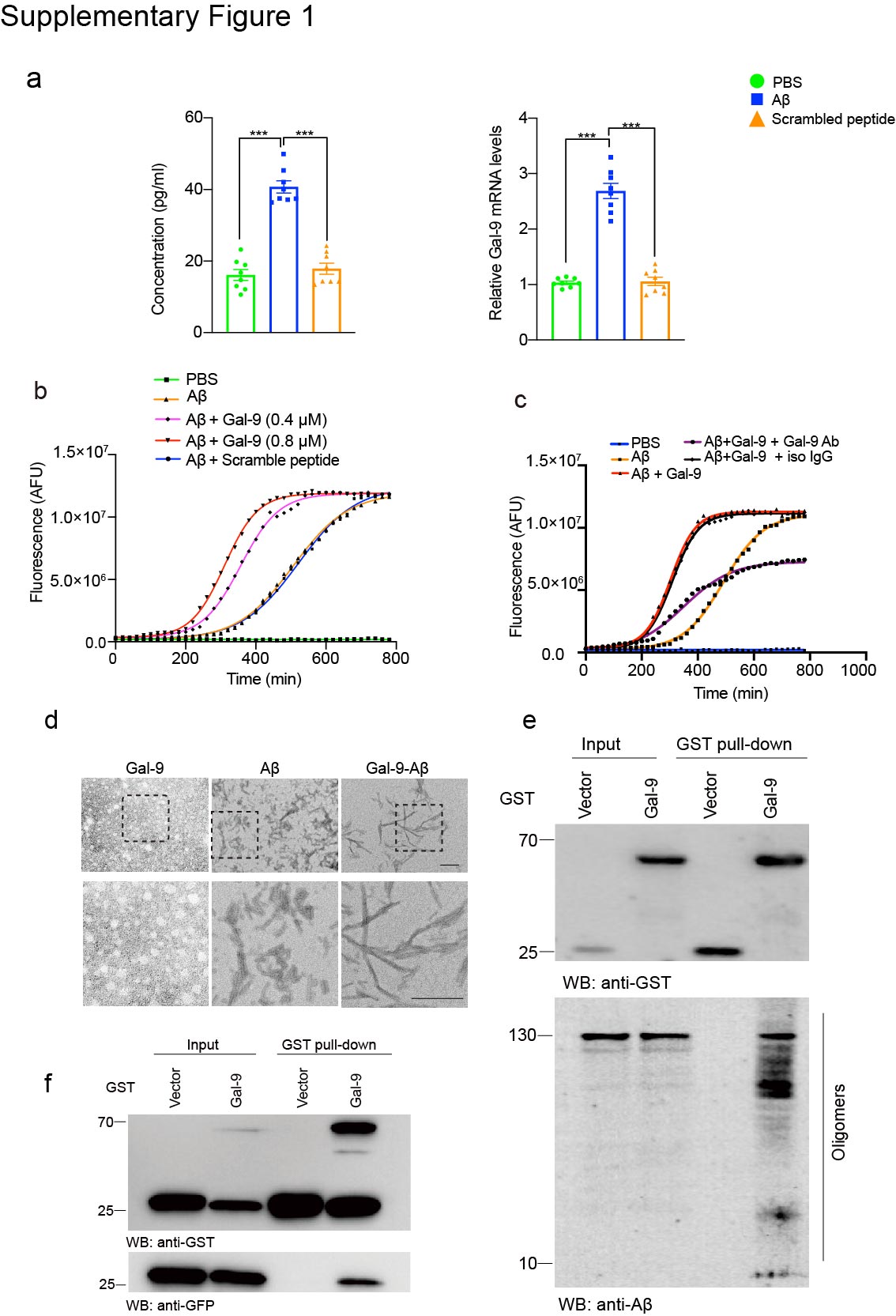

Supplement: Supplementary file 1 — Figure S1. [file ACEL-24-e14396-s003.jpg]

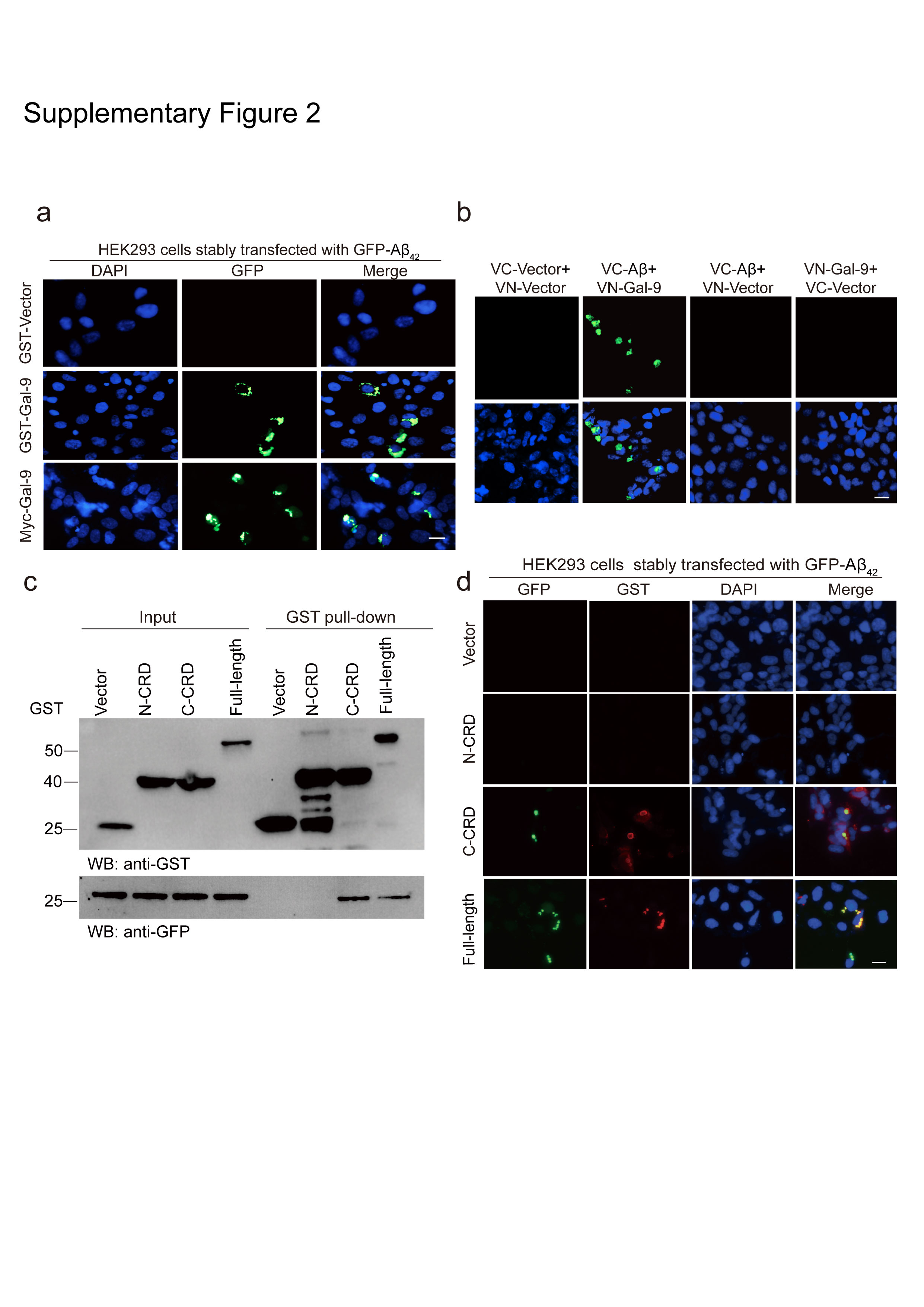

Supplement: Supplementary file 2 — Figure S2. [file ACEL-24-e14396-s006.jpg]

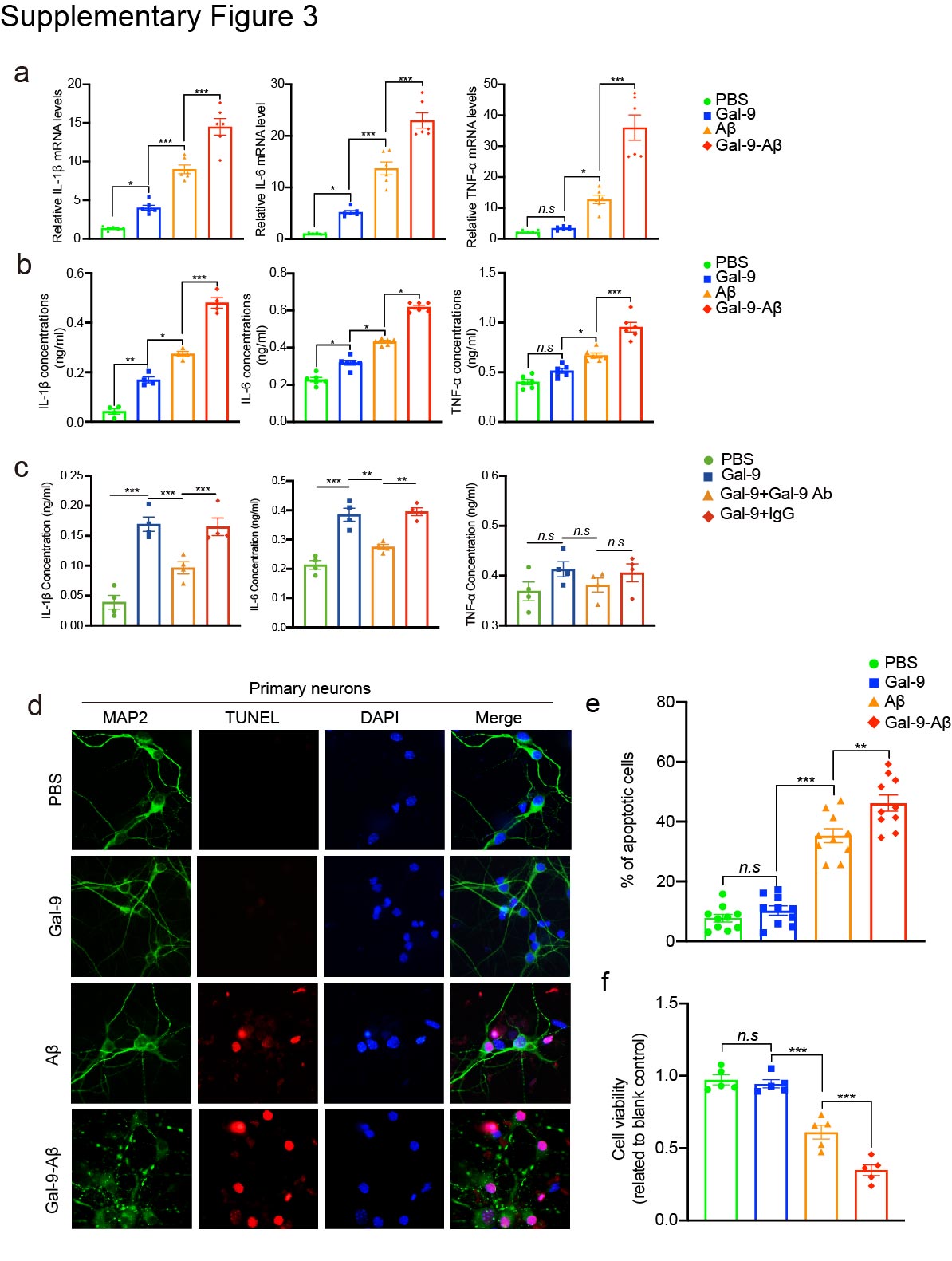

Supplement: Supplementary file 3 — Figure S3. [file ACEL-24-e14396-s004.jpg]

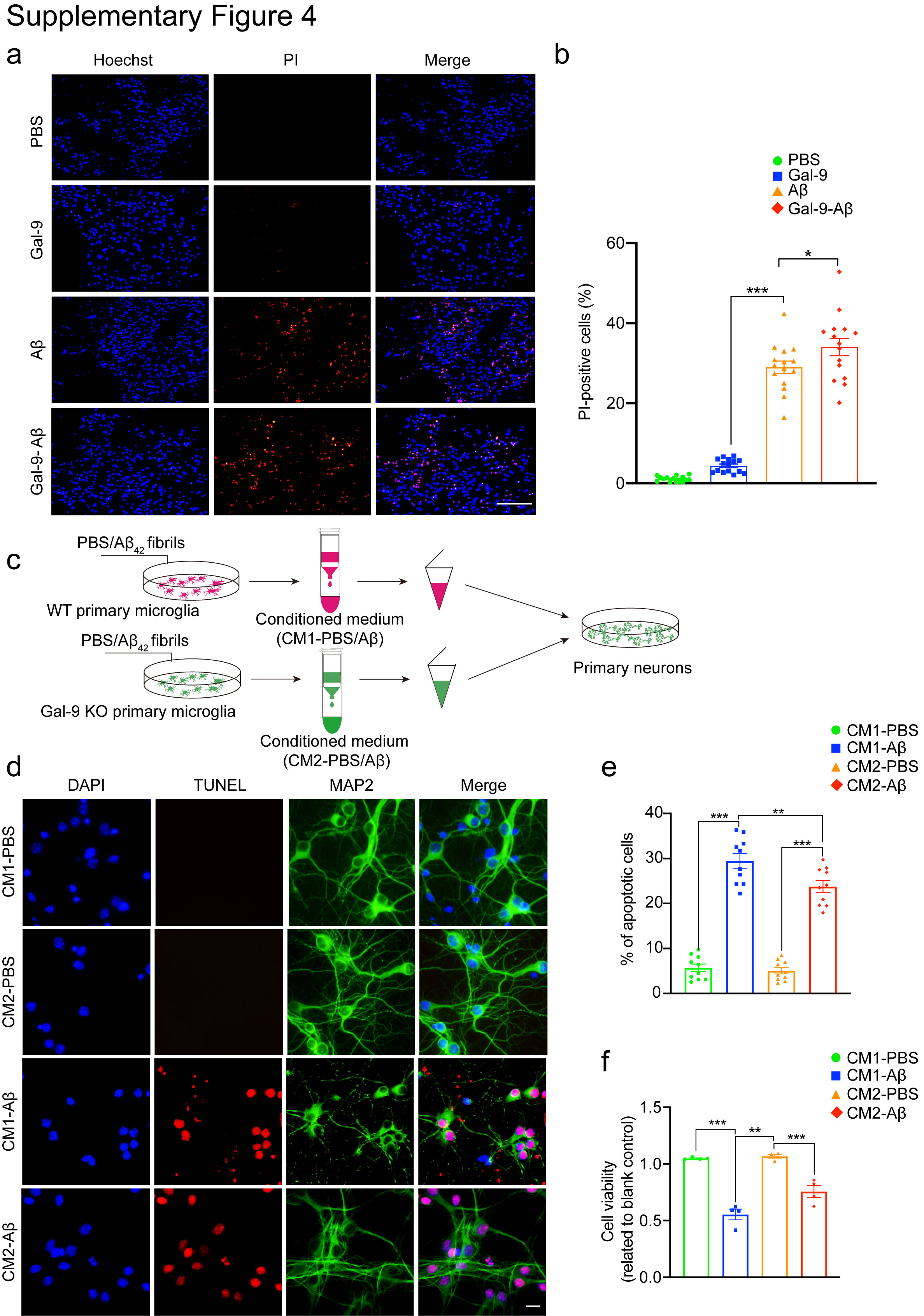

Supplement: Supplementary file 4 — Figure S4. [file ACEL-24-e14396-s007.jpg]

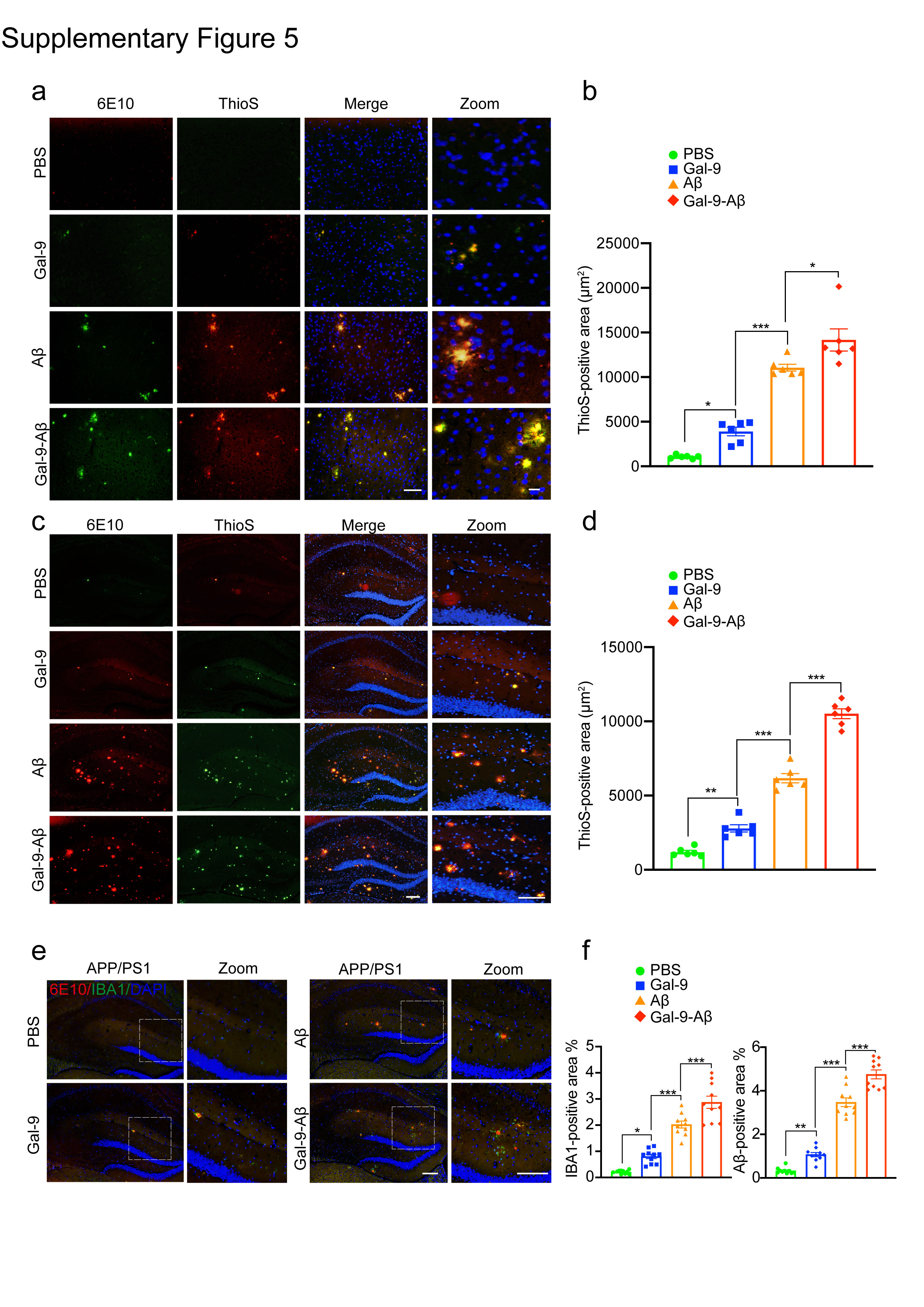

Supplement: Supplementary file 5 — Figure S5. [file ACEL-24-e14396-s001.jpg]

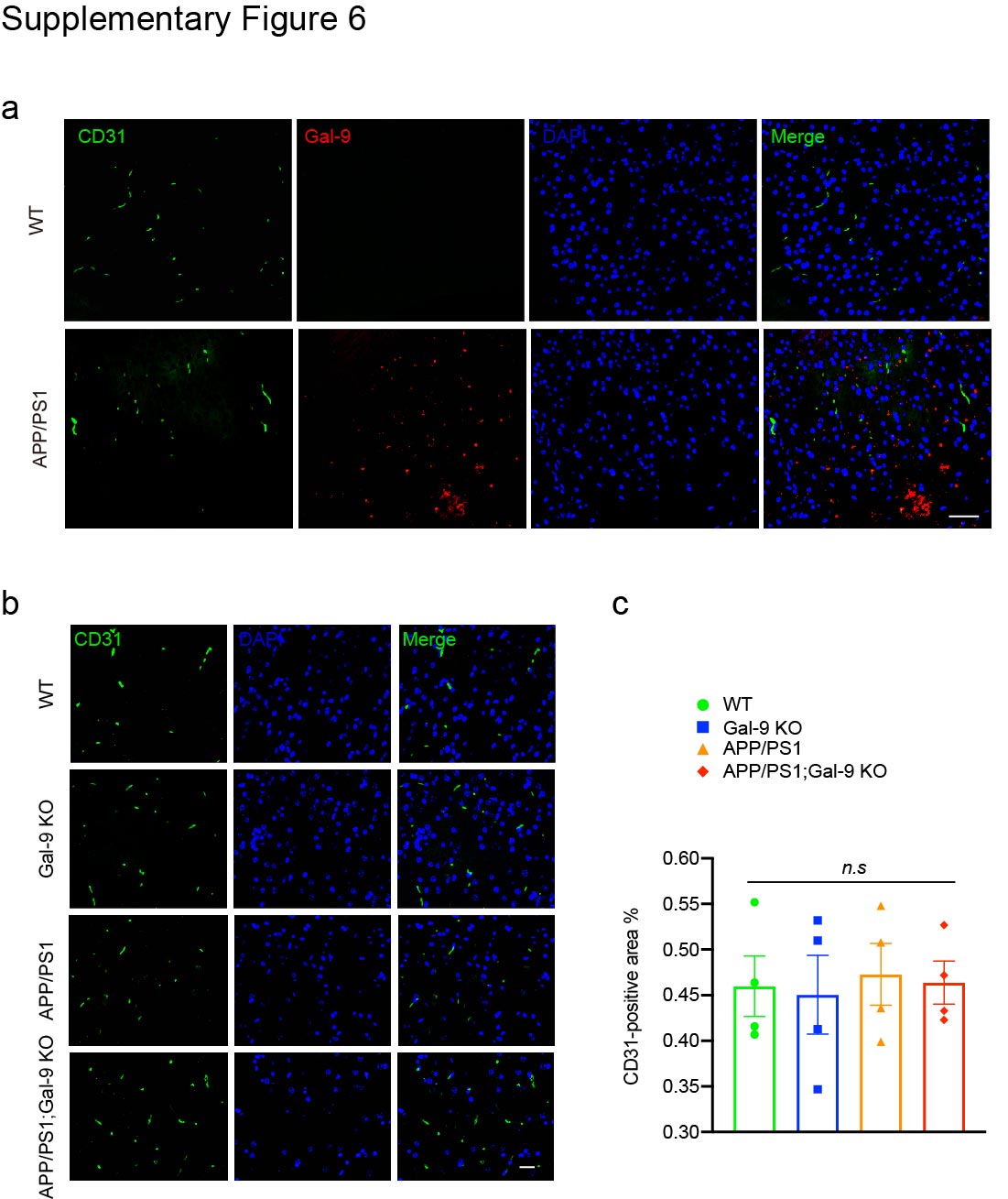

Supplement: Supplementary file 6 — Figure S6. [file ACEL-24-e14396-s005.jpg]

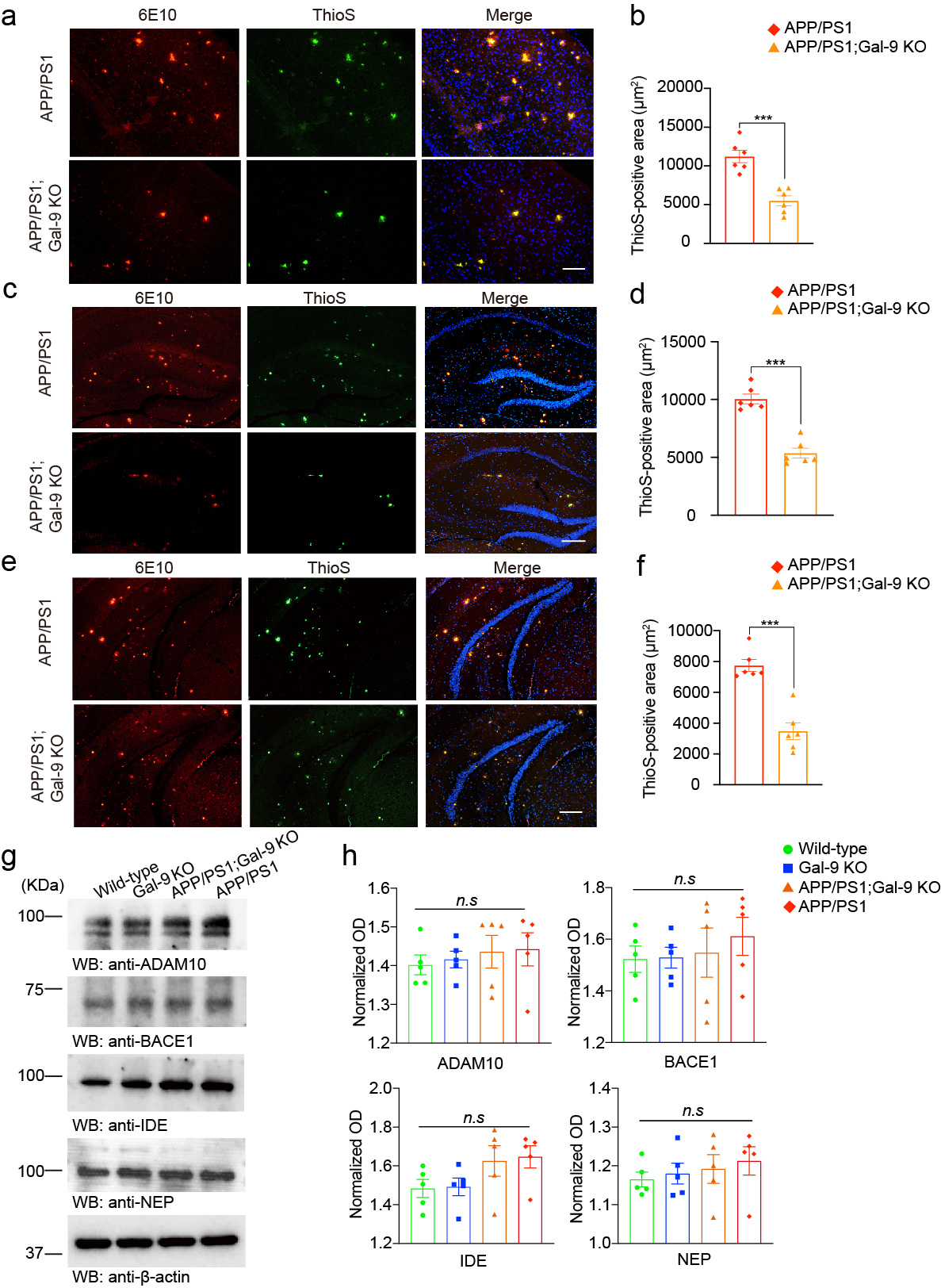

Supplement: Supplementary file 7 — Figure S7. [file ACEL-24-e14396-s008.jpg]
