# Supplementary material for: Microglia‐derived Galectin‐9 drives amyloid‐β pathology in Alzheimer's disease
Source: Aging Cell. 2024 Nov 1;24(2):e14396. doi: 10.1111/acel.14396 (PMC11822670; doi:10.1111/acel.14396)
Supplement: Supplementary file 8 — Table S1. [file ACEL-24-e14396-s009.docx]

**Table S1. Clinical information of human post-mortem tissues in Fig. 1a, 1c, and 1g.**

| Group | Age at death | Sex | PMD (hr) | Braak stage |
| --- | --- | --- | --- | --- |
| AD | 70 | F | 7 | IV |
|  | 65 | M | 9 | III |
|  | 78 | F | 17 | V |
|  | 91 | F | 21 | VI |
|  | 84 | F | 34 | VI |
|  | 78 | M | 19 | V |
|  | 85 | F | 14 | VI |
|  | 82 | M | 8 | V |
|  | 73 | M | 10 | IV |
|  | 79 | F | 9 | V |
| Control | 75 | M | 16 | - |
|  | 77 | F | 11 | - |
|  | 76 | M | 13 | - |
|  | 78 | F | 7 | - |
|  | 82 | F | 10 | - |
|  | 67 | M | 6 | - |
|  | 89 | F | 12 | - |
|  | 92 | F | 18 | - |
|  | 78 | M | 13 | - |
|  | 75 | M | 21 | - |

PMD, post-mortem delay.
